# A method for expressing two proteins at a tunable ratio allows for studying the kinetics of creatine transport by MCT12

**DOI:** 10.1016/j.isci.2026.117719

**Published:** 2026-09-30

**Authors:** Danila Boytsov, Julia Bicher, Oliver Kudlacek, Klaus Schicker, Rodion Tsurikov, Sonja Sucic, Michael Freissmuth, Harald H. Sitte, Walter Sandtner, Marko Roblek

**Affiliations:** 1 Institute of Pharmacology, Center for Physiology and Pharmacology, Medical University of Vienna, Waehringerstr. 13a, 1090 Vienna, Austria; 2 Hourani Center for Applied Scientific Research, Al-Ahliyya Amman University, Amman 19328, Jordan; 3 Center for Addiction Research and Science, Medical University of Vienna, 1090 Vienna, Austria

**Keywords:** tunable protein co-expression, SLC characterization, creatine transporter, kinetic modeling, genetic fit algorithm

## Abstract

Interactions between physically or functionally coupled proteins are often studied by co-expression from the same or separate plasmids, but protein ratios can vary widely between individual cells. To overcome this limitation, we developed a strategy to generate tunable and constant expression ratios using a single vector encoding two proteins separated by a P2A site followed by short AAA codon repeats that progressively reduce ribosomal readthrough. After validating this approach with the fluorescent proteins eGFP and mCherry, we applied it to characterize the transport cycle of the electroneutral monocarboxylate transporter MCT12, a task that is otherwise difficult to achieve experimentally. By co-expressing MCT12 with the concentrative creatine transporter CRT1 at defined ratios, we were able to determine the substrate turnover rate of MCT12 and its substrate K_M_. This approach enables precise control of protein stoichiometry, facilitating quantitative mechanistic studies of interacting proteins in cells.

## Highlights

• The described method enables precise control of protein expression ratios
• Protein expression ratios remain constant within cells of the same population
• Functional characterization of the electroneutral creatine transporter MCT12
• Method for the study of functionally and physically interacting proteins

## Graphical abstract

**Figure:**
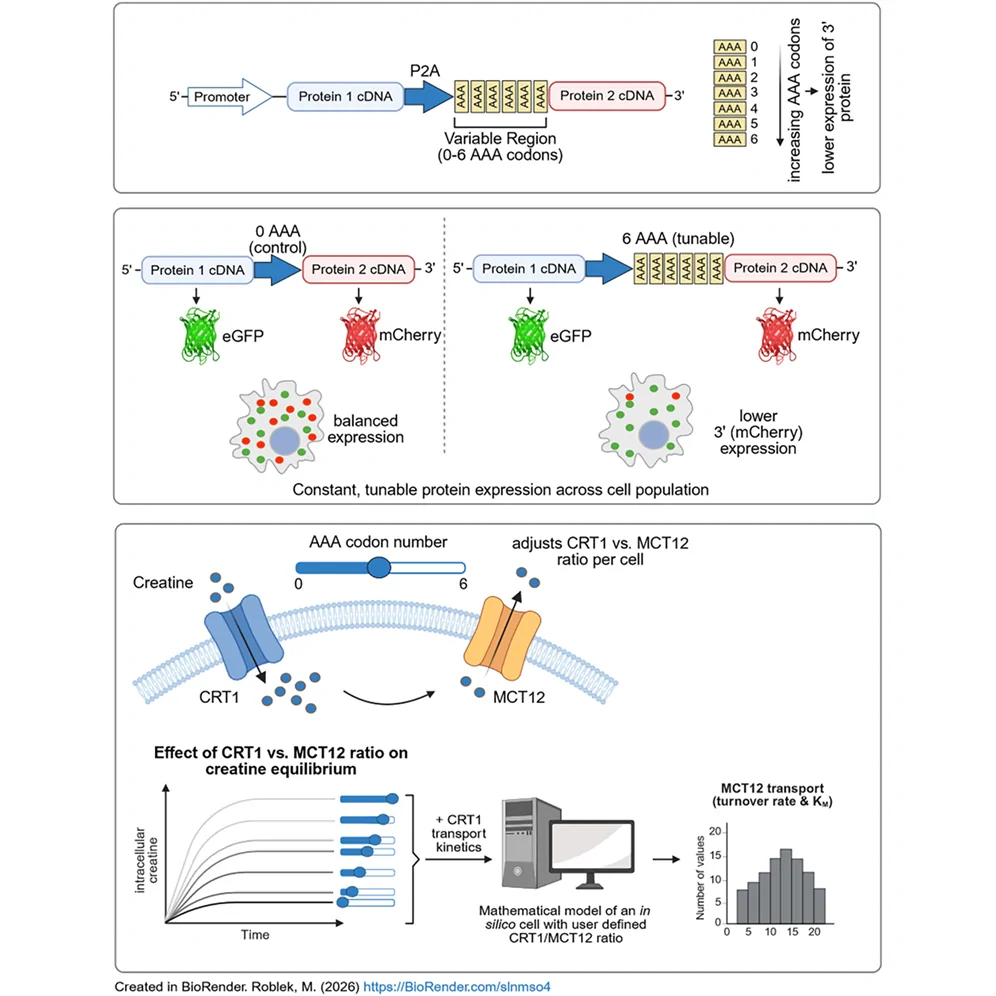

## Teaser

Protein; Molecular biology; Biophysics

## Introduction

Heterologous expression of proteins was introduced more than four decades ago^1^ and has since been a pillar in studying their function in a cellular context. In most instances, only one protein is overexpressed either constitutively or in an inducible manner. However, the study of interacting proteins, protein complexes, and multi-subunit proteins requires the co-expression of multiple proteins, and this ideally ought to be achieved at controllable ratios. Methods for co-expressing more than one protein rely on (1) co-transfection of different vectors, (2) inducing expression from bicistronic vectors equipped with either an internal ribosome entry site (IRES) or a Porcine teschovirus-1 2A (P2A) site, or (3) vectors with multiple promoters. Co-transfection of two or more plasmids typically leads to variable expression ratios between individual cells.^2^^,^^3^^,^^4^ In addition, different plasmid ratios can be variably affected by resource limitation.^5^ Expression from bicistronic vectors or vectors with multiple promoters results in a constant protein ratio across cells, but this ratio cannot be varied. Here, we report a method for co-expression at adaptable protein ratios, which we tested using two protein pairs: (1) eGFP and mCherry and (2) creatine transporter 1 (CRT1/SLC6A8) and the monocarboxylate transporter 12 (MCT12/SLC16A12). Our method utilizes the self-cleaving P2A sequence^6^ in combination with increasing triple-A codons inserted between the nucleotide sequences of the genes of interest. An increasing number of AAA codons leads to reduced expression of proteins downstream of the AAA codons.

In the present study, we validated this approach and used it to study the interplay between CRT1 and MCT12. Both carriers transport creatine, a key component of energy metabolism. The two transporters have different transport mechanisms: CRT1 is a secondary active transporter,^7^ while MCT12 operates as a uniporter.^8^ They are co-expressed in neuronal cells and other cell types where they are predicted to operate together to control the intracellular creatine levels.^9^

The transport kinetics of CRT1 have been studied in detail, but much less is known about the activity of MCT12. The patch-clamp technique is uniquely suited to investigate the transport kinetics of solute carriers because of its unrivaled temporal resolution and because it allows for the control of all intrinsic parameters that govern the function of transporters (i.e., membrane voltage, the composition of the extracellular and intracellular solutions, and temperature). However, substrate flux through MCT12 does not give rise to a transport-associated current because of the zwitterionic nature of creatine and due to the fact that it is a uniporter.

Here, we present a new method that we used to determine the transport kinetics of MCT12. We conducted uptake experiments on cells expressing CRT1 and MCT12 at constant ratios and measured intracellular creatine concentration. The results from these measurements were then analyzed using a mathematical model of a cell expressing the two transporters. Through this model, we estimated the substrate turnover rate of MCT12 in both the forward and reverse directions, as well as the corresponding K_M_ values by leveraging our knowledge of the transport kinetics of CRT1. This was achieved by employing a genetic fitting algorithm to search for suitable model parameters that were able to explain the experimentally observed intracellular and extracellular creatine concentrations in cells that expressed both transporters at varying ratios.

## Results

### Achieving tunable expression ratios of two proteins that are constant across cells

We designed two constructs: (1) a vector encoding 5′ mCherry followed by 3′ eGFP (Figure 1A) and (2) an eGFP and mCherry in the 5′ and 3′ positions, respectively (Figure 1D). In both constructs, the 5′ and 3′ coding sequences were interrupted by an intervening sequence coding for the self-cleaving P2A site to allow for the expression of two proteins from one open reading frame. Downstream of the P2A site, we inserted up to six AAA triplets in frame with the codons of the proteins. The AAA codon codes for the amino acid lysine (K). Therefore, we used the notation K_n_ in the following description, with the subscript “n” indicating the number of inserted AAA triplets.

**Figure 1:**
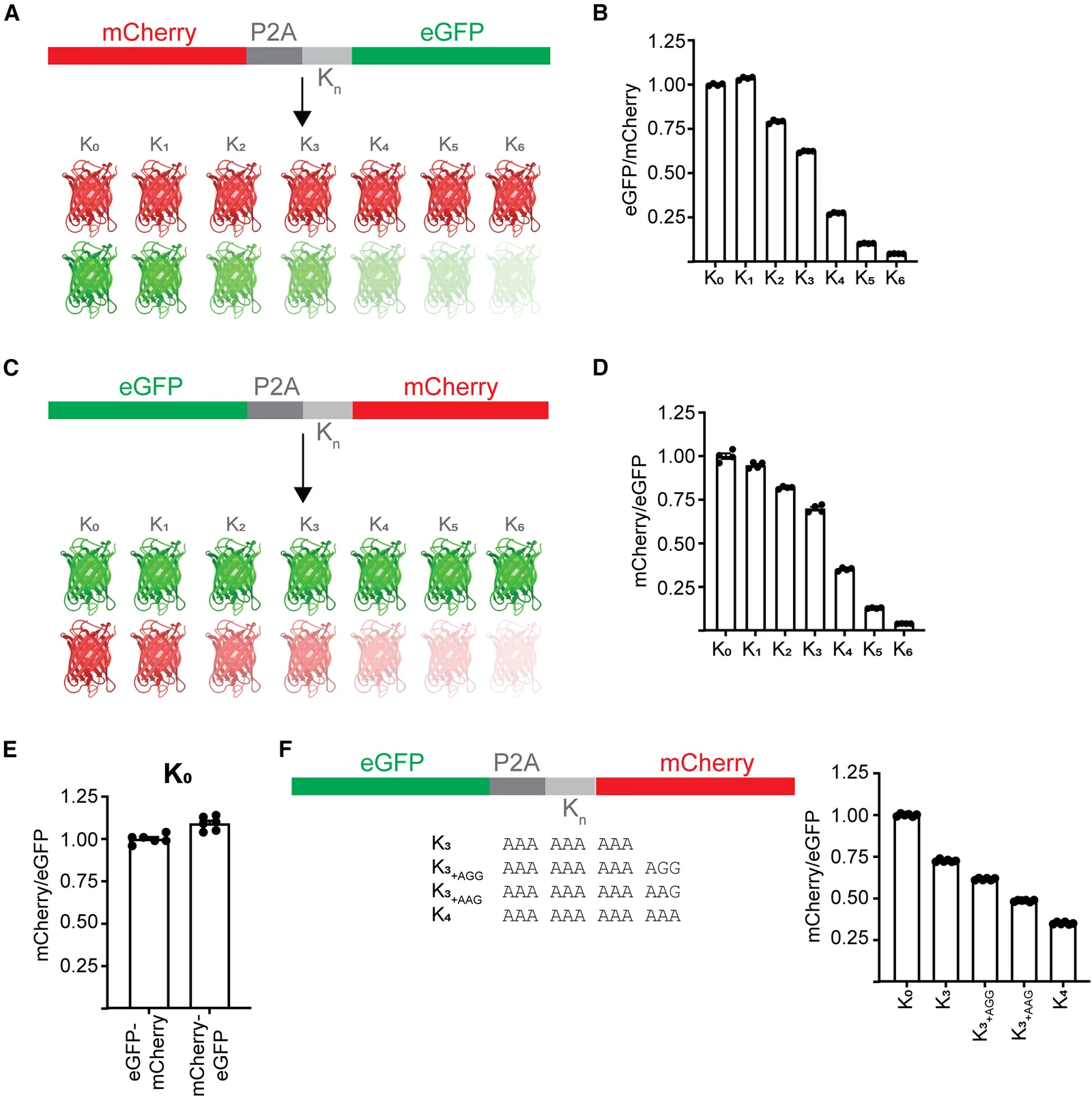
Adaptable expression ratios of two proteins from one open reading frame (A) Scheme of the mCherry-P2A-K_n_-eGFP construct. K_n_ indicates the number of AAA codons, coding for the amino acid lysine (K). Expected relative expression levels are shown in the lower image. Increasing levels of transparency indicate reduced expression of eGFP. (B) Ratio of eGFP and mCherry determined by flow cytometry (*n* = 4). Data are represented as mean ± SEM. (C) Scheme of the eGFP-P2A-K_n_-mCherry construct. Expected relative expression levels are shown in the lower image. (D) Ratio of mCherry and eGFP expression determined by flow cytometry (*n* = 4). Data are represented as mean ± SEM. (E) Flow cytometry analysis of mCherry-P2A-K_0_-eGFP and eGFP-P2A-K_0_-mCherry expressing cells. The ratio of mCherry to eGFP expression is shown for both constructs (*n* = 6). Data are represented as mean ± SEM. (F) Scheme of the eGFP-P2A-K_n_-eGFP construct with the K_n_ nucleotide sequence indicated in the lower image. The ratio of mCherry and eGFP expression determined by flow cytometry is shown on the right (*n* = 6). Data are represented as mean ± SEM. See also Figure S1.

The expression of mCherry-P2A-K_n_-eGFP in HEK293 cells was induced with 1 μg/mL doxycycline 24 h prior to flow cytometry analysis. A representative fluorescence-activated cell sorting (FACS) plot shows that all cells were transfected (Figure S1A). We found consistent expression of mCherry (the N-terminal protein) across all K_n_ variants (Figure S1B), but a decrease in eGFP expression as the number of AAA codons increased (Figures 1B and S1B). The most pronounced drop in eGFP expression was observed between K_3_ and K_4_ (eGFP/mCherry = 0.62 to 0.27; eGFP/mCherry was set to 1.00 for the K_0_ construct where no AAA triplet had been inserted). In the K_6_ variant, this ratio dropped to 0.04.

Earlier reports posited that the mRNA downstream of a poly-AAA codon was enzymatically degraded^10^^,^^11^ and that this explained the reduced expression of the protein following the poly-A codon. We tested this hypothesis by analyzing the mRNA levels by qPCR. Our results showed indistinguishable eGFP/mCherry mRNA ratios between the K_0_ and K_6_ variants (Figure S1C). This finding refutes the assumption that the progressive reduction in eGFP expression with increasing number of inserted AAA codons is the result of mRNA degradation.

We also tested constructs in which we had swapped the position of eGFP and mCherry (i.e., eGFP-P2A-K_n_-mCherry, see Figure 1C). Again, the expression of the 5′ protein (in this case eGFP) was similar across the K_n_ variants (Figure S1D), but there was a gradual decrease in mCherry expression with increasing number of AAA codons (Figures 1D and S1D). As with the other set of constructs, we found the most pronounced decline in mCherry expression between K_3_ and K_4_ (mCherry/eGFP = 0.70 to 0.35; again, the mCherry/eGFP ratio was set to 1.00 for the K_0_ construct). The mCherry/eGFP mRNA ratios between K_0_ and K_6_ variants remained indistinguishable (Figure S1E).

In the K_0_ variants (mCherry-P2A-K_0_-eGFP and eGFP-P2A-K_0_-mCherry), both proteins are expected to be expressed at a 1:1 ratio due to the absence of AAA codons following the P2A site. We verified this assumption by determining the mCherry/eGFP ratio using flow cytometry for both K_0_ variants. Our results showed that the mCherry/eGFP ratio was similar between the two constructs (Figure 1E). The equal ratio confirms that the 5′ and 3′ terminal proteins are expressed at a 1:1 ratio.

The significant drop in the expression level of the C-terminal protein between the K_3_ and K_4_ constructs (Figures 1B and 1D) prompted us to test if we could achieve intermediate expression levels by replacing the fourth AAA triplet with the triplets AGG (coding for the amino acid arginine) or AAG (coding for the amino acid lysine) (Figure 1F). We inserted these codons into eGFP-P2A-K_n_-mCherry and expressed the resulting constructs in HEK293 cells. As seen, the incremental increase in the number of adenine nucleotides yielded variants that displayed intermediate mCherry levels (mCherry/eGFP for K_3_ = 0.73, K_3+AGG_ = 0.62, K_3+AAG_ = 0.49, and K_4_ = 0.35; again, the mCherry/eGFP was set to 1.00 for the K_0_ construct, Figure 1F).

Stretches of positively charged amino acids reduce the translation efficiency of ribosomes.^12^ We explored whether the effect on the expression level is mediated by the AAA codons or by the positive charge of the encoded lysines by replacing all AAA codons with those codons specifying arginine (AGG). We measured the mCherry levels in eGFP-P2A-K_6_-mCherry, eGFP-P2A-R_6_-mCherry (all lysines replaced by arginine), and, as a control, in eGFP-P2A-A_6_-mCherry (all lysines replaced by alanine) using flow cytometry. We observed the strongest reduction of mCherry expression with the eGFP-P2A-K_6_-mCherry construct (mCherry/eGFP = 0.04), supporting the idea that the stretch of AAA codons has the most pronounced effect on downstream translation efficiency. However, while the six alanine codons had no effect (mCherry/eGFP = 1.06), inserting six arginine codons also hampered mCherry translation, but to a lesser extent (mCherry/eGFP = 0.44) (Figure S1F), suggesting an additive effect of positively charged amino acids.

Finally, we tested whether an out-of-frame premature stop in mCherry translation is responsible for the reduction of mCherry expression in eGFP-P2A-K_6_-mCherry. This was addressed by inserting either one or two additional nucleotides before the mCherry nucleotide sequence to offset a potential frameshift caused by the six AAA codons. Flow cytometry analysis revealed no rescue in the expression of mCherry (mCherry/eGFP = 0.009 for K_6+1_ and 0.02 for K_6+2_) (Figure S1G). Accordingly, it is unlikely that a frameshift is the cause of the loss in mCherry expression.

### Tunable expression ratios of the creatine transporters CRT1 and MCT12

We exploited our approach to address the interplay of CRT1 and MCT12. HEK293 cells endogenously express CRT1 at low levels. Because of the very large concentrative power of CRT1,^9^ this low endogenous expression suffices to support substantial accumulation of [^3^H]creatine over time (Figure S2A). Accordingly, we created a HEK293 SLC6A8^−/−^ cell line by CRISPR-Cas9-based deletion to obviate any confounding effect in the subsequent analysis: in these SLC6A8^−/−^ cells, [^3^H]creatine uptake was abolished (Figure S2A).

CRT1^3xHA^-P2A-K_n_-MCT12^2xmyc^ constructs (Figure 2A) were expressed in HEK293 SLC6A8^−/−^ cells, and the expression levels were determined by immunoblotting (Figure 2B). The expression of MCT12^2xmyc^ gradually decreased as the number of AAA triplets increased (Figure 2C). Moreover, the drop in the expression of the second protein (MCT12) was similar to that seen for the constructs encoding the fluorescent proteins. In further agreement, we found indistinguishable mRNA levels of the 3′ MCT12^2xmyc^ in the K_0_ and K_6_ variants (Figure S2C).

**Figure 2:**
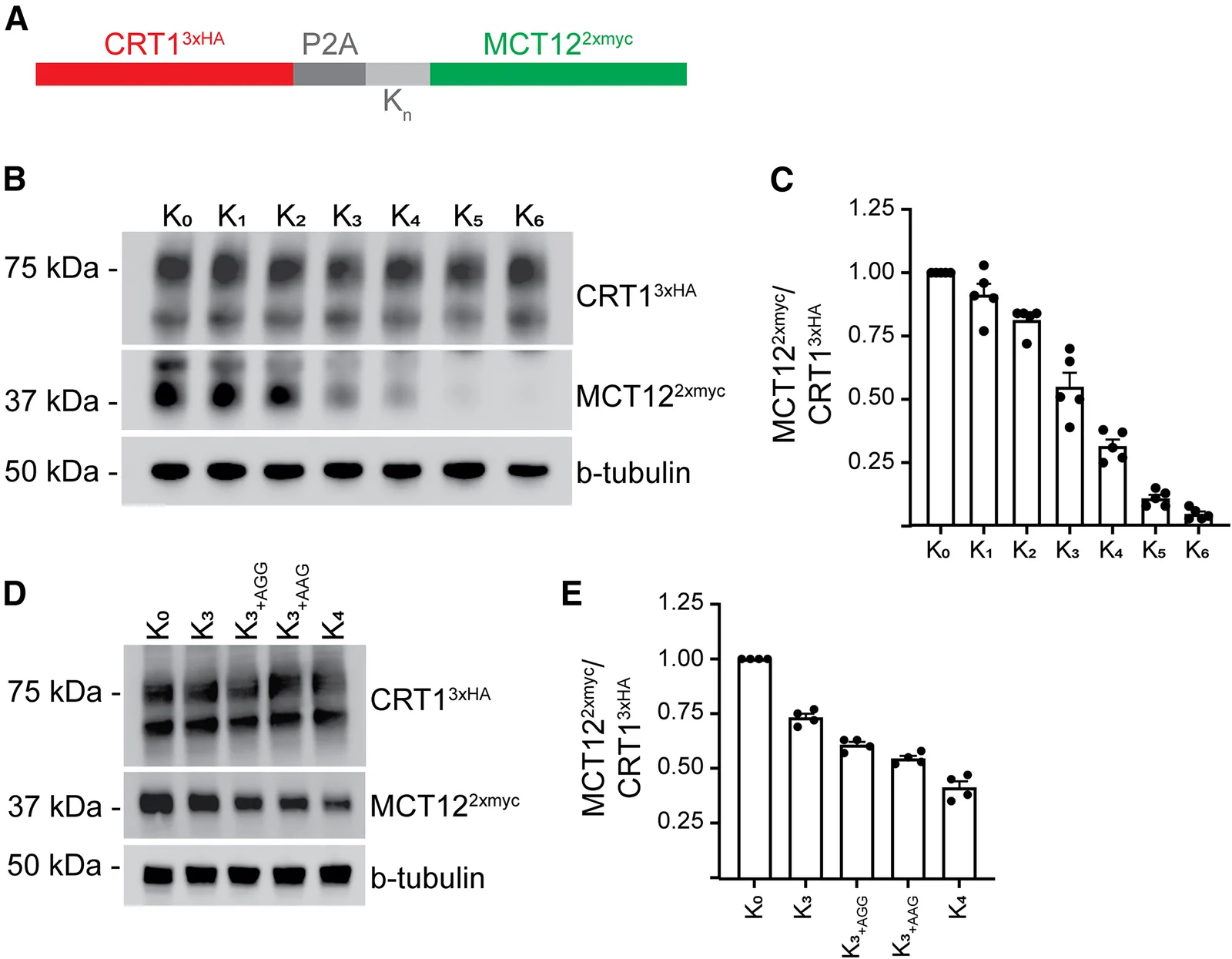
Adaptable expression ratios of the creatine transporters CRT1 and MCT12 in HEK293 SLC6A8^−/−^ cells (A) Scheme of the CRT1^3xHA^-P2A-K_n_-MCT12^2xmyc^ construct. (B) Immunoblot analysis of CRT1^3xHA^-P2A-K_n_-MCT12^2xmyc^. (C) Ratio of MCT12^2xmyc^ and CRT1^3xHA^ expression as determined by immunoblotting (*n* = 5). Data are represented as mean ± SEM. (D) Immunoblot analysis of CRT1^3xHA^-P2A-K_n_-MCT12^2xmyc^. (E) Ratio of MCT12^2xmyc^ and CRT1^3xHA^ expression as determined by immunoblotting (*n* = 4). Data are represented as mean ± SEM. See also Figure S2.

Similar to the pair of fluorescent proteins (cf. Figure 1F), we obtained intermediate expression levels of CRT1^3xHA^ and MCT12^2xmyc^ in the K_3_ and K_4_ variants (Figures 2D and 2E). In addition, we confirmed that the six lysines in front of MCT12^2xmyc^ in the CRT1^3xHA^-P2A-K_6_-MCT12^2xmyc^ construct did not function as a degron, because incubation of the cells in the presence of bafilomycin A and/or MG132 only resulted in a modest increase in the amount of MCT12^2xmyc^ (Figure S2D). This observation indicates that there is no functional ribosomal readthrough in the K_6_ variant.

In the immunoblots, we observed two prominent bands for CRT1^3xHA^. In contrast, MCT12^2xmyc^ migrated as a single band. This indicates differences in the extent of glycosylation for the two transporters. We treated the CRT1^3xHA^-P2A-K_0_-MCT12^2xmyc^ cell lysate with two deglycosylating enzymes, PNGaseF and endoglycosidase (EndoH). We identified the lower molecular weight band of CRT1^3xHA^ as the immature core-glycosylated variant by its sensitivity to EndoH. The higher molecular weight band of CRT1^3xHA^ was the mature glycosylated variant because it was sensitive to PNGaseF but not to EndoH. In contrast, migration of MCT12^2xmyc^ was not affected by either deglycosylating enzyme, indicating that the glycan content of MCT12^2xmyc^ is modest at best (Figure S2E). Furthermore, we did not detect any CRT1^3xHA^-P2A-K_n_-MCT12^2xmyc^ fusion proteins, indicating very efficient peptide bond skipping by the P2A site (Figure S2F).

We determined the relative expression level of the two transporters by tagging CRT1 and MCT12 with eGFP and mCherry, respectively. We analyzed the following two constructs using flow cytometry: CRT1^eGFP^-P2A-K_0_-MCT12^mCherry^ and MCT12^mCherry^-P2A-K_0_-CRT1^eGFP^ (Figure 3A). Surprisingly, CRT1^eGFP^-P2A-K_0_-MCT12^mCherry^ produced about twice as much CRT1^eGFP^ as MCT12^mCherry^ (mCherry/eGFP = 0.50). However, when the positions of the two genes were swapped (MCT12^mCherry^-P2A-K_0_-CRT1^eGFP^), both transporters were equally expressed (mCherry/eGFP = 1.03) (Figure 3B).

**Figure 3:**
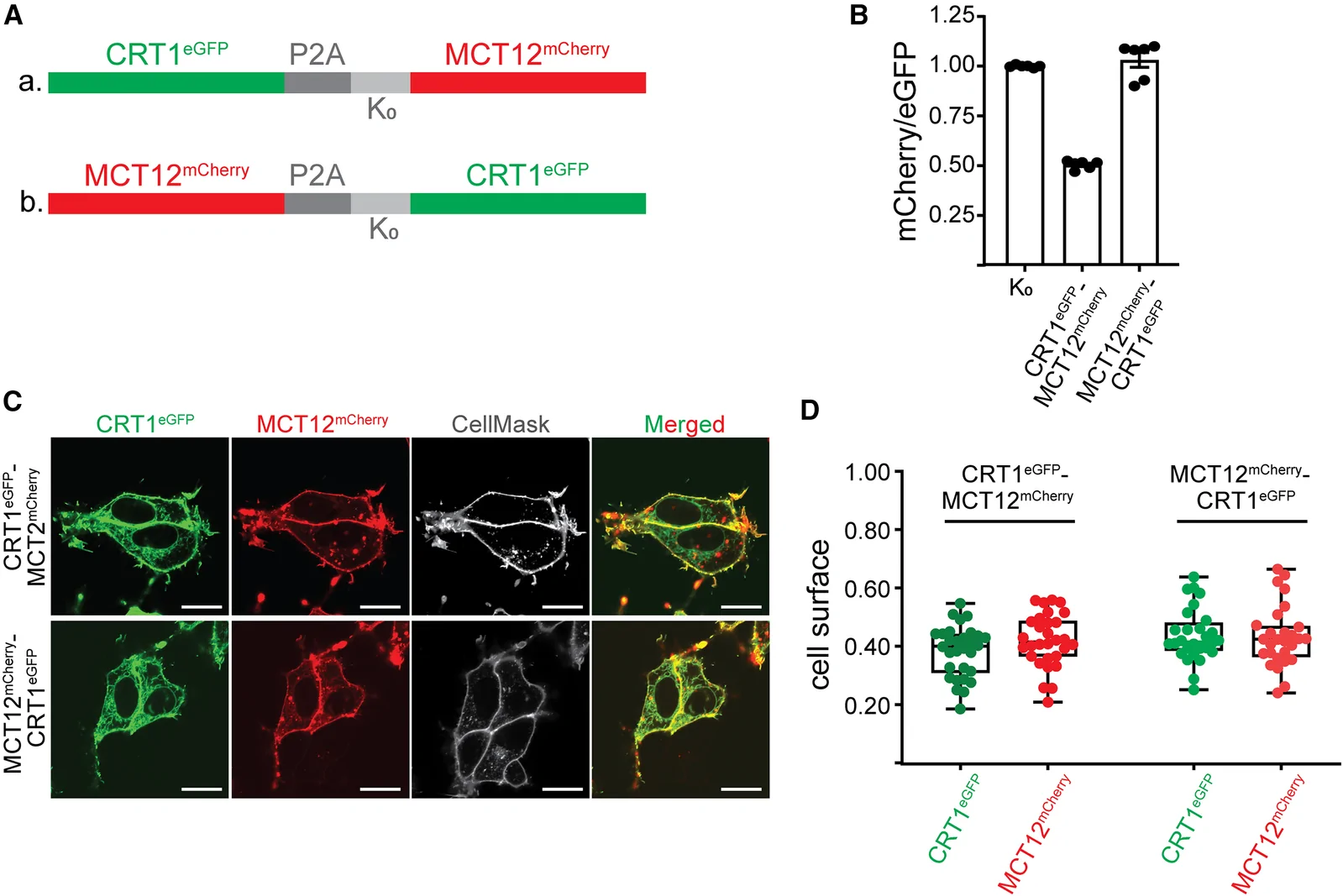
Relative cell surface expression of the creatine transporters CRT1 and MCT12 in HEK293 SLC6A8^−/−^ cells (A) Scheme of the CRT1^eGFP^-P2A-K_0_-MCT12^mCherry^ (a) and MCT12^mCherry^-P2A-K_0_-CRT1^eGFP^ construct (b). (B) Ratio of mCherry and eGFP expression in eGFP-P2A-K_0_-mCherry (K_0_, as a control), CRT1^eGFP^-P2A-K_0_-MCT12^mCherry^, and MCT12^mCherry^-P2A-K_0_-CRT1^eGFP^ constructs as determined by flow cytometry (*n* = 6). Data are represented as mean ± SEM. (C) Live-cell confocal microscopy of HEK293 SLC6A8^−/−^ cells expressing CRT1^eGFP^-P2A-K_0_-MCT12^mCherry^ and MCT12^mCherry^-P2A-K_0_-CRT1^eGFP^. The plasma membrane was stained with CellMask Deep Red. Scale bars, 20 μm. (D) Analysis of cell surface expression of CRT1^eGFP^ and MCT12^mCherry^. *n* = 30 (CRT1^eGFP^-P2A-K_0_-MCT12^mCherry^); *n* = 28 (MCT12^mCherry^-P2A-K_0_-CRT1^eGFP^).

In order for CRT1 and MCT12 to be functional, they must reach the surface membrane and not be trapped in subcellular locations. Therefore, we used confocal microscopy to gauge the percentage of CRT1^eGFP^ and MCT12^mCherry^ correctly trafficked to the cell surface. Regardless of whether CRT1^eGFP^ or MCT12^mCherry^ was the N- or C-terminal protein, the fraction on the cell surface for both proteins was the same (around 40%) (Figures 3C and 3D).

### Interplay between CRT1 and MCT12

We recorded currents from a K_0_ cell co-expressing CRT1 and MCT12 using the whole-cell patch-clamp technique (Figure 4A). When 1 mM of creatine was applied, a substrate-induced current was detected in the presence of extracellular Na^+^, but not when Na^+^ was replaced by the inert cation N-methyl-D-glucamine (NMDG^+^). Previous studies have shown that CRT1 relies on the Na^+^ gradient for the transport of creatine, while MCT12 does not.^8^^,^^13^ Hence, in the absence of Na^+^, only MCT12 is active. However, creatine transport through MCT12 did not generate a current. This is anticipated due to the zwitterionic nature of creatine and because MCT12 operates as a uniporter.

**Figure 4:**
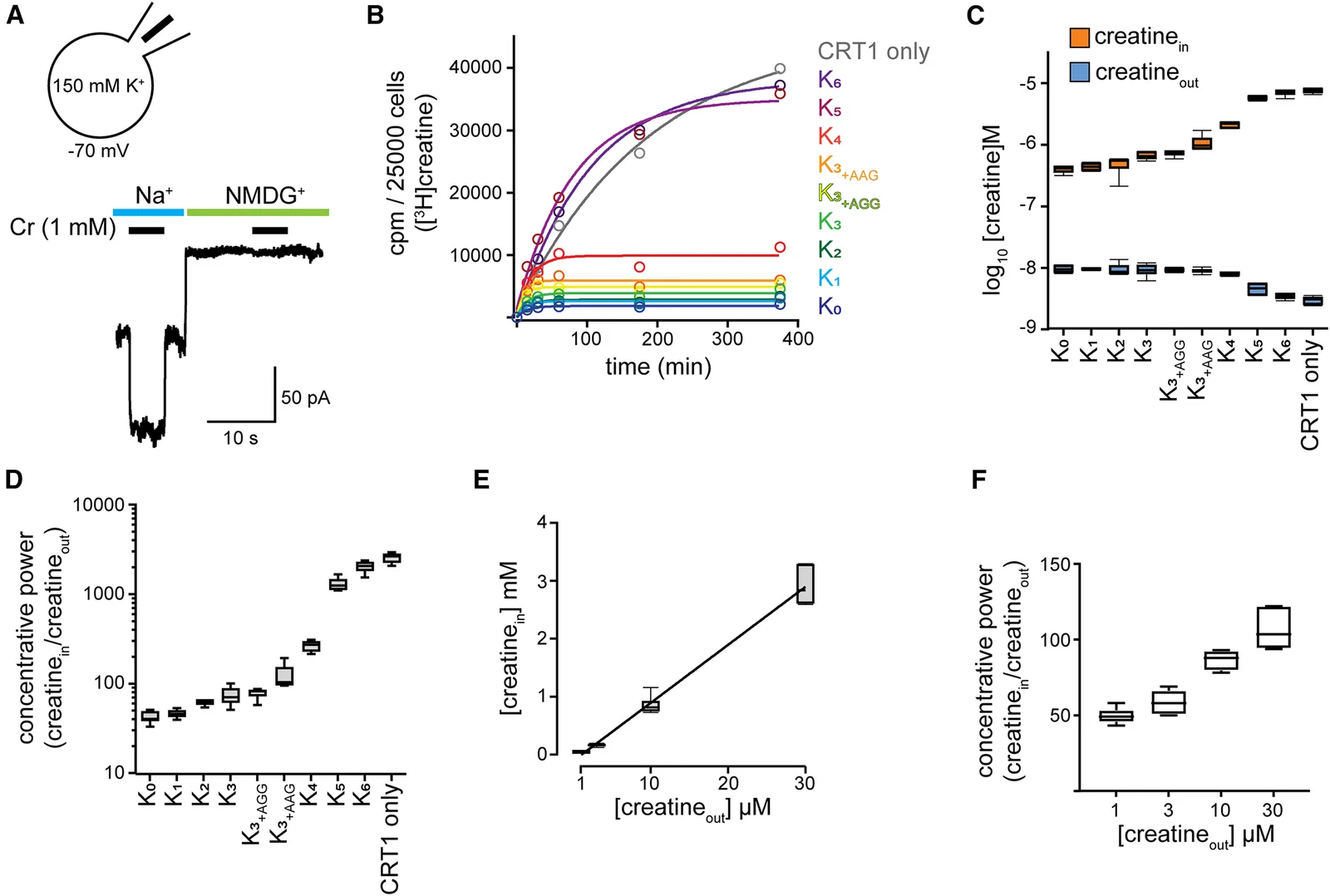
Creatine uptake by the K_n_ variants (A) Patch-clamp recording obtained from a K_0_ cell. In the presence of extracellular Na^+^ (150 mM), the application of 1 mM creatine resulted in an inwardly directed current carried by CRT1. This current was absent when Na^+^ was replaced by NMDG^+^. Shown is a representative current trace. (B) Time-dependent uptake of [^3^H]creatine (10 nM) into cells of the K_n_ variants and cells expressing only CRT1. One representative experiment of six is shown (cpm = counts per minute). (C) Extracellular and intracellular creatine concentrations at steady state in the K_n_ variants (*n* = 6). Data are represented as mean ± SD. (D) Concentrative power (i.e., creatine_in_/creatine_out_) of the K_n_ variants calculated from the data shown. Data are represented as mean ± SD. (E) Steady-state creatine concentrations measured in K_0_ cells when challenged with 1, 3, 10, and 30 μM creatine. In these experiments, the specific activity of [^3^H]creatine was progressively diluted by addition of unlabeled creatine (*n* = 6). Data are represented as mean ± SD. (F) The concentrative power of K_0_ cells was calculated from the data shown and plotted as a function of the extracellularly applied creatine concentration. Data are represented as mean ± SD.

We performed radioligand uptake experiments to monitor the intracellular accumulation of creatine over time in cells expressing CRT1 and MCT12 at varying ratios (K_0_ to K_6_) and in cells that only express CRT1 (Figure 4B). Creatine uptake was measured after 15, 30, 60, 175, and 375 min following exposure to 10 nM [^3^H]creatine at 37°C. As the relative expression levels of MCT12 per CRT1 decreased, the intracellular creatine levels rose and reached equilibrium more slowly. The most parsimonious explanation for the inverse relationship between MCT12 expression and the intracellular creatine concentration is that creatine taken up by CRT1 can exit the cells via MCT12.

The outer and inner concentrations of creatine at steady state were calculated from the results obtained from six uptake experiments similar to the one shown in Figure 4B. From K_4_ cells upwards, the outer concentration significantly decreased (Figure 4C). This depletion occurred because these cells accumulated a large fraction of the available creatine. From these data, we calculated the concentrative power (creatine_in_/creatine_out_) of the tested constructs (Figure 4D). Depending on the relative levels of CRT1 and MCT12 expression, the concentrative power reached values between 45 (for K_0_) and 2,600 (for cells that only expressed CRT1). We also measured intracellular creatine concentration at steady state within K_0_ cells exposed to 1, 3, 10, and 30 μM extracellular creatine (Figure 4E). Interestingly, the concentrative power of the K_0_ cells rose when challenged with higher creatine concentrations (Figure 4F).

The tested constructs (CRT1^3xHA^-P2A-K_n_-MCT12^2xmyc^) expressed CRT1 as the N-terminal protein, which unavoidably results in a non-native N terminus of MCT12 containing the remaining proline from the P2A site, as well as an increased number of lysine residues. To investigate a potential influence of the N-terminal modification on MCT12 function, we measured creatine uptake in HEK293 cells expressing MCT12^mCherry^-P2A-K_0_-CRT1^eGFP^ (Figure S3). In this construct, the N terminus of MCT12 remains unmodified. Our analysis shows that the N-terminal modification does not affect MCT12 function.

### Analysis of transporter kinetics

We aimed to obtain kinetic information on the electrically silent MCT12 by leveraging our knowledge of the transport kinetics of CRT1 and the intracellular creatine concentrations at steady state measured in cells expressing both transporters at varying ratios. The approach required the development of a mathematical model of an *in silico* cell that expresses CRT1 and MCT12 at user-defined ratios on its surface (for model details see supplemental information). We recently parameterized a kinetic model of CRT1 using data obtained from patch clamp experiments.^9^ However, our previous measurements were conducted at room temperature (i.e., 25°C), but the current uptake experiments were all performed at 37°C. We addressed the disparity between datasets by first assessing how temperature affects the transport kinetics of CRT1. Accordingly, we measured creatine-induced currents in the same cell at 25°C, 30°C, and 37°C (Figure 5A). Raising the temperature resulted in an increase in the current through CRT1. The normalized current amplitudes are displayed in an Arrhenius plot (Figure 5B). Additionally, we examined the temperature dependence of K_M_ of CRT1 for creatine by measuring currents through CRT1 at various creatine concentrations at both 25°C and 37°C. The normalized current amplitudes plotted against the applied creatine concentration revealed a modest rightward shift of the substrate K_M_ at the higher temperature (Figure 5C).

**Figure 5:**
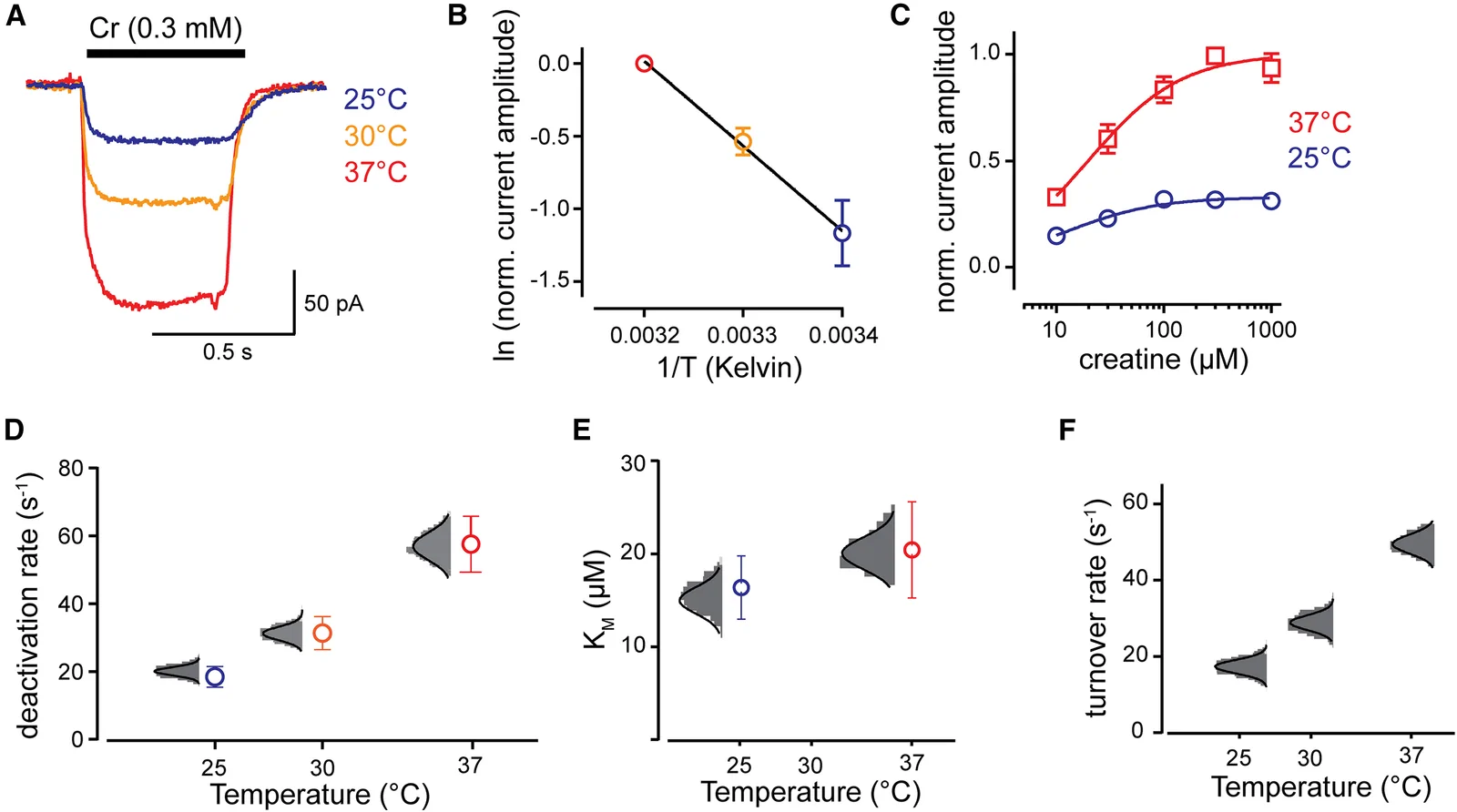
Temperature dependence of CRT1 (A) CRT1-mediated currents recorded from the same cell at 25°C, 30°C, and 37°C. (B) Arrhenius plot of the normalized current amplitudes (*n* = 6). Data are represented as mean ± SD. (C) Normalized current amplitudes as a function of the creatine concentration measured at 25°C and 37°C (*n* = 6). The lines in the graph are fits of the Michaelis-Menten curve to the data points. The K_M_ values estimated by the fit were 15.5 ± 0.14 μm at 25°C, and 20 ± 0.37 μM at 37°C. Data are represented as mean ± SD. (D) Current deactivation rates as a function of temperature. Plotted are the experimentally determined average values ±SD, calculated from experiments as performed in (A), as well as the histograms of the corresponding predicted values obtained from 1,000 fitting runs. (E) K_M_ values of CRT1 for creatine transport at 25°C and 37°C obtained from experiments and the histograms of the predicted values from 1,000 fitting runs. Data are represented as mean ± SD. (F) Turnover rate of CRT1 at 25°C, 30°C, and 37°C extracted by the model from the experimental data.

We used a genetic fitting algorithm to adjust the kinetic model of CRT1 to match the normalized current amplitudes determined at different creatine concentrations and temperatures (Figures 5B and 5C). The fitting process seeks suitable model parameters by minimizing the sum of squared residuals between the prediction of the model and the experimental data. We relied on the Eyring equation to formulate a model that can accommodate temperature changes (description of the model, available at https://doi.org/10.5281/zenodo.2274524).

Automating the search for model parameters has an additional advantage: it allows for the computation of the range of model outputs resulting from experimental uncertainties. This represents a step forward from previous approaches that only provided single model outcomes without offering insights into how experimental uncertainties affect them. We globally fit the model to normalized current amplitudes. Each amplitude is described by its average value and an associated standard deviation. Instead of fitting only the average values in a single fitting run, we conducted multiple runs where, in each run, we randomly selected amplitudes from normal distributions defined by the experimentally determined average values and standard deviations. This bootstrapping approach allowed for obtaining ranges of values for the descriptors of CRT1-mediated transport (e.g., substrate K_M_ and the substrate turnover rate) along with their probability distribution.

We aimed to build a model that can account for both average values and experimental uncertainties of observables. One such observable is the current deactivation rate (i.e., the rate at which the current vanished upon removal of creatine from the bath solution), which accelerated as the temperature was raised. The deactivation rate is an imprecise proxy of the substrate turnover rate.^14^ Its average value and uncertainty were determined at 25°C, 30°C, and 37°C: it is evident from Figure 5D that the model was able to reproduce not only the average values but also the experimental uncertainties. This was also true for the K_M_ values determined at 25°C and 37°C (Figure 5E). The probability distributions of the substrate turnover rates of CRT1 at 25°C, 30°C, and 37°C as extracted by the model are also shown (Figure 5F). However, the turnover rate is a model prediction and not directly observable.

In the following step, we aimed to parameterize a kinetic model of MCT12 using the data obtained from the creatine uptake experiments, where we measured the creatine concentrations in the K_n_ variants at equilibrium. It is important to note that the creatine concentrations at equilibrium depend on the relative, not the absolute, expression levels of the two transporters. Absolute levels only affect how fast the equilibrium is reached. In the same K_n_ variant, the relative expression levels of CRT1 and MCT12 remain constant across cells. This is not necessarily true for absolute expression levels. For example, in the experiment depicted in Figure 4B, the K_6_ cells initially accumulated creatine at a faster rate than the cells only expressing CRT1, indicating a higher number of transporters on the surface of K_6_ cells. However, eventually the creatine concentration in cells only expressing CRT1 surpassed that in the K_6_ cells since they lacked MCT12 expression.

We parameterized the reaction scheme of MCT12 (Figure 6A) by searching for a set of rate constants that can account for the observed inner and outer concentrations of creatine at steady state (Figures 4C and 4E). For this, we employed the same bootstrapping method as described previously. The other necessary parameters in the model of the *in silico* cell are the rate constants of CRT1 at 37°C (description of the model, available at https://doi.org/10.5281/zenodo.2274524). However, instead of using a single set of rate constants, we utilized a thousand sets obtained from fitting the normalized current amplitudes of CRT1. In each of the fitting runs, we randomly selected one of the thousand sets, with each set having an equal probability of being chosen.

**Figure 6:**
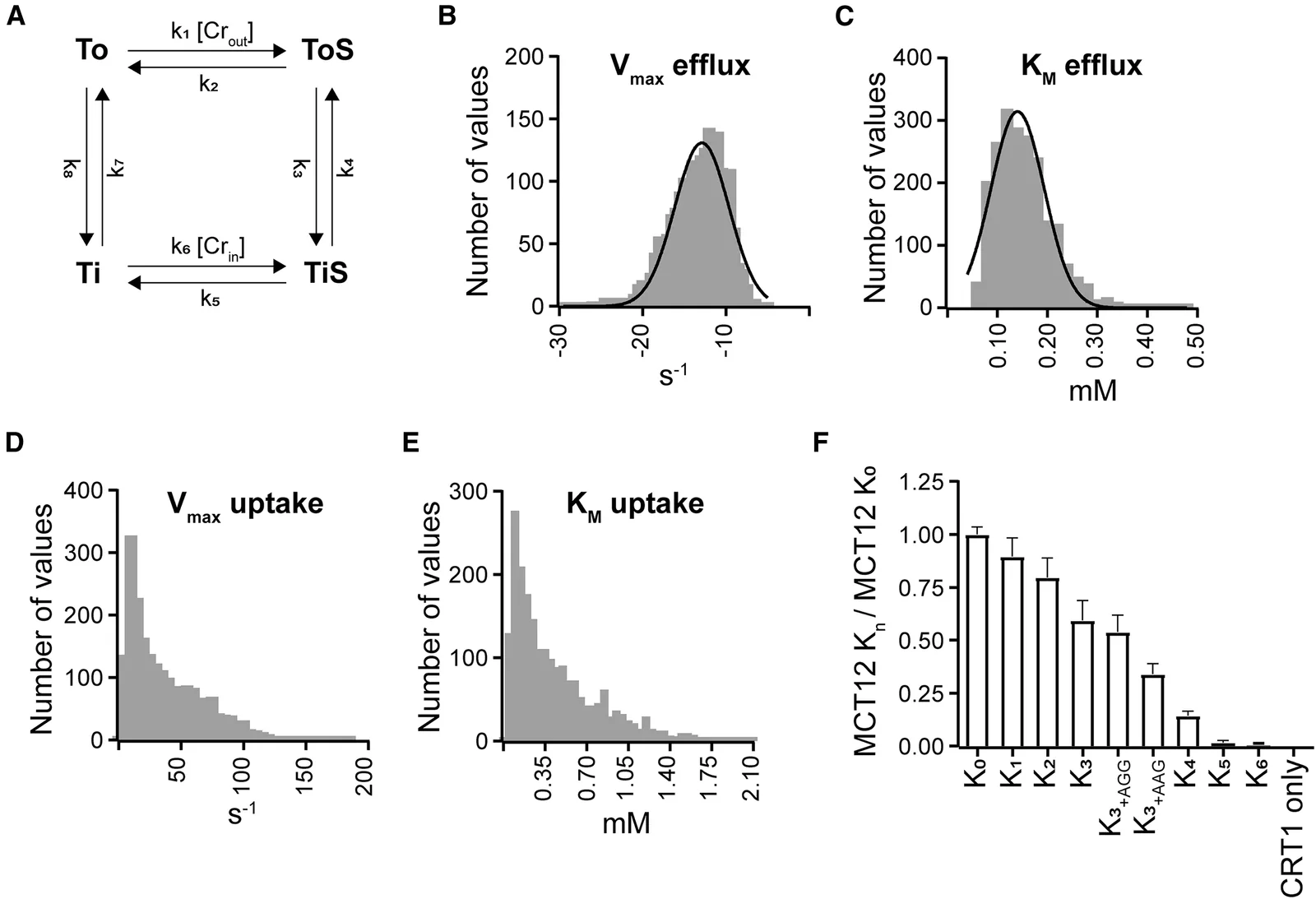
Transporter kinetics of MCT12 (A) Reaction scheme of the transport cycle of MCT12. (B) Histogram of the creatine turnover rate of MCT12 in the efflux direction. Negative numbers indicate the rate in the efflux direction. (C) Histogram of the creatine K_M_ of MCT12 in the efflux direction. (D) Histogram of the creatine turnover rate of MCT12 in the uptake direction. (E) Histogram of the creatine K_M_ of MCT12 in the uptake direction. The histograms in (B, C, D, and E) represent the corresponding values obtained from 2,000 fitting runs. The black lines in (B and C) represent fits of a normal distribution to the data points (V_max_ efflux = −12.9 ± 3.3 s^−1^, K_M_ efflux = 0.14 ± 0.05 mM). (F) Expression of MCT12 in the K_n_ variants relative to the expression in K_0_ cells, calculated by the model. Data are represented as mean ± SD.

The reaction scheme in Figure 6A is that of an equilibrative transporter defined by eight rate constants, all of which are voltage independent in the case of MCT12. The genetic fitting algorithm identifies values for these rate constants that explain the experimentally determined inner and outer creatine concentrations inside and outside cells of the K_n_ variants. However, the values for the rate constants obtained from individual fitting runs varied widely, indicating that these parameters are non-identifiable (description of the model, available at https://doi.org/10.5281/zenodo.2274524). This was not the case for the descriptors of MCT12-dependent transport. As MCT12 is an equilibrative transporter, these descriptors include the substrate turnover rates in the forward and reverse directions (creatine uptake and efflux, respectively) as well as the corresponding K_M_ values. We determined their values using a method originally described by King and Altman,^15^ which we previously employed to derive algebraic equations for the descriptors of transport function based on the underlying rate constants.^16^ These equations are listed in the description of the model, available at https://doi.org/10.5281/zenodo.2274524.

The output of the model is a range of (plausible) rate constants for each partial reaction of the transport cycles of CRT1 and MCT12 (description of the model, available at https://doi.org/10.5281/zenodo.2274524). The rate constants, which were derived for MCT12, were used to compute the distribution of the values for V_max efflux_, K_M efflux_, V_max_ _uptake_, and K_M__uptake_ of MCT12: it is evident that the values of V_max efflux_ (Figure 6B) and K_M efflux_ (Figure 6C) have narrower and more symmetric distributions than V_max_ _uptake_ (Figure 6D) and K_M__uptake_ (Figure 6E). However, it is also evident from Figures 6D and 6E that, in spite of the skewed distribution, there is a sharp peak for the estimates of both V_max_ _uptake_ and K_M__uptake._ The corresponding estimates (with a V_max_ _uptake_ and a K_M__uptake_ of approximately 15 s^−1^ and 0.15 mM, respectively) are equivalent to the corresponding estimates for V_max efflux_ and K_M efflux_ defined by the peaks of the distribution shown in Figures 6B and 6C, respectively. The model also estimates the relative expression levels, i.e., the ratio of MCT12 over CRT1 at any given K_n_ (*n* = 0 to 6) and additional insertions (e.g., K_3+AGG_ or K_3+AAG_). The estimates of these ratios (Figure 6G) were in excellent agreement with the expression ratios determined by immunoblotting (cf. Figures 2B–2E). This provided an independent validation of the model.

## Discussion

In a cellular context, proteins operate in a network of direct and indirect interactions. Their expression levels also vary in a cell type-specific manner. The strength of these interactions is predicted to differ accordingly. Thus, tools that allow for fine-grained adjustment of expression ratios are desirable as a starting point for interrogating the interrelation of two proteins. In this study, we designed a simple and versatile method to express two proteins at tunable ratios, which are constant across cells of the same population, and exploited this method to obtain kinetic information on creatine transport by the electrically silent, equilibrative transporter MCT12. Our observations provide the following insights and advances: (1) up to six AAA-sites suffice to cover a large range of ratios in a tunable manner. This was verified with both a set of soluble proteins (i.e., GFP and RFP) and a set of membrane proteins (i.e., CRT1 and MCT12). In addition, and importantly, we show that the tunable ratio was maintained regardless of the order in which the cDNAs were inserted. Thus, the principle is expected to be broadly applicable. (2) The approach can be used to address the kinetic properties of efflux transporters, which are otherwise difficult to determine. (3) We developed a genetic algorithm, which allows for a realistic evaluation of kinetic parameters and allows for selecting the most accurate parameters over a broad range of possibilities.

We demonstrate that six AAA codons (K_6_) separating two protein-coding sequences are sufficient to cause a reduced expression of the C-terminal sequence of only 4%, when compared to the no-insert (K_0_) constructs. We did not investigate the effect of additional insertions, because our current approach covered a range of 25:1 to 1:25. We consider this range to be adequate to address the vast majority of relevant biological questions pertaining to direct and indirect interactions of proteins in a cellular context. In addition, and importantly, the insertion of a variable number of triple A-repeats allows for interrogating the interaction by changing the relative abundance of the interaction partners. Finally, further adjustments in the protein ratios can readily be achieved by extending the stretch of adenine nucleotides by using codons with a single A (i.e., K_3+AGG_) or double A (i.e., K_3+AAG_). This results in smaller decrements and, thus, an even more fine-grained tuning. A previous report found that an insertion of up to 20 AAA-codon repeats between coding sequences was required to reduce expression of the 3′ gene product.^11^^,^^17^ The discrepancy between these earlier observations and our findings could lie in the construct design. In Arthur et al.,^11^ the stalling sequence appeared shortly after translation start, while in Juszkiewicz et al.^17^ it was between two P2A sites flanked by eGFP and mCherry. Thus, the position of the stalling sequence in relation to the translation start might have influenced the efficiency of ribosome stalling. A previous report showed that ribosome stalling involves different biological mechanisms depending on whether it occurs during initial or later stages (elongation phase) of translation.^18^ In addition, unlike in the previous studies, we used stable cell lines with doxycycline-inducible expression of the constructs.

Several hypotheses exist as to explain why repeats of AAA triplets impact protein translation. These include a decline in mRNA stability,^11^ ribosome sliding leading to frameshift,^10^ the presence of the encoded lysines, which act as a secondary degron motif,^19^^,^^20^ and ribosome stalling.^17^^,^^21^^,^^22^ Our experiments were not primarily designed to address these controversies. However, the amount of mRNA encoding the 3′ gene product did not differ in the K_0_ and K_6_ variants (Figures S1C and S1E), which argues against an effect on mRNA stability. The decline in levels of the protein encoded by the 3′ mRNA was not remedied by inserting one or two nucleotides (Figure S1G). This finding refutes the idea that AAA repeats cause a frameshift, which leads to no-go-mediated or nonsense-mediated mRNA degradation. In addition, we did not find any evidence for enhanced proteolytic degradation of the downstream protein when this harbored 6 lysines on its N terminus (Figure S2D). Instead, our data are consistent with a model positing that the AAA repeats progressively impede ribosomal readthrough (i.e., they cause ribosome stalling) because they engage nucleotides of rRNA in the ribosomal decoding center through a π-interaction. This interaction prevents tRNA from occupying the decoding center by maintaining the AAA triplets in a decoding-incompetent conformation.^21^^,^^22^ As an alternative explanation, the ε-amino group of the encoded lysines has been proposed as the main cause of decreased expression of the downstream protein: in this model, electrostatic interactions between the ribosome tunnel and stretches of positively charged amino acids in the nascent peptide cause ribosomal stalling and, thus, reduce translation efficiency.^23^ However, in the K_6_ variant, the downstream protein output decreased 25-fold and in the R_6_ variant only 2-fold (Figure S1F). Based on this observation, we conclude that these electrostatic interactions have only a weak effect on protein translation.

We capitalized on our ability to maintain constant protein ratios to collect kinetic information on creatine transport by MCT12. Cells, which rely on ATP-buffering by phosphocreatine, maintain the required high free creatine levels through uptake by CRT1.^24^ In our cellular model system (i.e., HEK293 cells overexpressing CRT1 and MCT12) steady-state creatine levels solely depend on the relative ratios of these two transporters, as *de novo* synthesis of creatine in HEK293 cells is absent^25^ and conversion to creatinine occurs at a negligible rate.^26^

We recently showed that, in neurons and striated muscles, CRT1 must work together with an efflux transporter to clamp the intracellular creatine concentration between 5 and 10 mM, i.e., at the physiological levels. We posited that MCT12 was this transporter, which served as the valve allowing for rapid equilibration of intracellular creatine^9^: the intracellular concentrations measured in neurons and muscle cells are roughly ten times lower than predicted from the creatine concentrations in the extracellular fluid (20–100 μM) and the concentrative power of CRT1 (see Figure 4). Here, we addressed the question whether creatine efflux through MCT12 was large enough to explain the discrepancy between the physiological concentrations of creatine within neurons and muscle cells and the levels predicted from the concentrative power of CRT1. The kinetics of the transport cycle of MCT12 are not readily accessible, because the transporter is electrically silent. In fact, consistent with a previous study,^27^ we failed to detect creatine influx upon heterologous expression of MCT12 in HEK293 cells. However, it was possible to explore the transport cycle of MCT12 in the presence of co-expressed CRT1. Our approach relies on the fact that the concentration at steady state solely depends on the relative expression ratio of CRT1 and MCT12, not their absolute levels. Thus, the creatine concentrations in cells expressing the K_n_ variants provided one set of crucial data. The other dataset was supplied by patch-clamp recordings of CRT1 to define the transport kinetics of CRT1 at 37°C (i.e., the temperature at which creatine uptake to steady state was determined). A mathematical model of an *in silico* cell, which expressed both transporters on its surface (at varying ratios), allowed for extracting the relevant parameters of the MCT12-mediated creatine influx and efflux. Our model computes comparable K_M_ values (about 0.15 mM) and turnover numbers (about 15 s^−1^) for inward and outward transport of creatine by MCT12. This is consistent with the equilibrative nature of MCT12-mediated creatine translocation. After expression of MCT12 in *Xenopus laevis* oocytes, higher K_M_ estimates (0.3–0.6 mM) were obtained for MCT12-mediated inward transport of creatine,^8^^,^^13^ but these data were obtained at 25°C and can, therefore, not be directly compared to our estimates. Regardless of these uncertainties, it is clear that under physiological conditions, MCT12 operates to support efflux of creatine: the extracellular creatine concentrations are in the range of 30–100 μM and of 17–90 μM in the periphery and in the brain, respectively.^28^ Intracellular levels are substantially higher, i.e., 7 and 5 mM in striated muscle^29^ and in neurons,^30^ respectively. Thus, the extracellular levels of creatine only result in modest occupancy of MCT12 in the outward-facing state. In contrast, the inward-facing state of MCT12 is essentially saturated by intracellular creatine. The formation of phosphocreatine from ATP and creatine by creatine kinase is energetically unfavorable.^31^ Hence, an excess of free creatine is required to support the ATP-buffering capacity and preclude accumulation of ADP. However, and surprisingly, in the myocardium, excessive accumulation of creatine is detrimental.^32^ Thus, creatine levels must be clamped within a range. It is, however, at present, not clear if the expression levels of MCT12 suffice to support the required efflux. In brain and in striated muscle, CRT1 transcripts are 40–100 times more abundant than those of MCT12.^33^ Hence, creatine efflux may be supported by additional identified creatine transporters, i.e., MCT9/SLC16A9,^13^ FliPT1/SLC22A15,^34^ or yet-to-be-identified solute carriers. In this context, it is noteworthy that HEK293 cells express low endogenous levels of both MCT9 and FliPT1 (Human Protein Atlas, proteinatlas.org). However, our estimates indicate that the endogenous expression of creatine-transporting SLCs is substantially lower than in overexpressing cells (Figure S2B) and, thus, immaterial for our analysis. Therefore, the present strategy of co-expressing these transporters together with CRT1 in HEK293 cells ought to be suitable for addressing their kinetic properties and their contribution to the maintenance of physiological levels of intracellular creatine.

### Limitations of the study

Possible limitations of the method described in this study depend largely on the nature of the biological question being addressed. One inherent feature of the approach is the unavoidable addition of up to six lysine residues to the N terminus of the downstream protein. Whether this modification affects the function of the second protein must, therefore, be assessed on a case-by-case basis. If such inference occurs, it may be possible to circumvent this problem by reversing the order of the genes. However, in the case of the bicistronic CRT1^3xHA^-P2A-K_n_-MCT12^2xmyc^ constructs, switching the gene order would likely have created a technical complication. Increased expression of MCT12 relative to CRT1 would foreseeably have reduced the intracellular creatine concentration to levels that can no longer be measured accurately. This example illustrates that the method does not suffer from universal limitations, but rather that its suitability must be evaluated in the context of each specific experimental application.

## Resource availability

### Lead contact

Requests for further information and resources should be directed to and will be fulfilled by the lead contact, Marko Roblek (marko.roblek@meduniwien.ac.at).

### Materials availability

All unique/stable reagents generated in this study are available from the lead contact with a completed materials transfer agreement.

### Data and code availability

• Raw data reported in this paper will be shared by the lead contact upon reasonable request.
• All original code and the description of the model have been deposited at Zenodo and are publicly available at https://doi.org/10.5281/zenodo.2274524 as of the date of publication.
• Any additional information required to reanalyze the data reported in this paper is available from the lead contact upon request.

## Acknowledgments

We thank the Flow Cytometry Facility at the Medical University of Vienna for their assistance with cell sorting. This research was funded by the 10.13039/501100002428Austrian Science Fund (10.13039/501100002428FWF), project P 36621-B (awarded to M.R.), and P 36667 (awarded to W.S.).

## Author contributions

Conceptualization, D.B., W.S., and M.R.; methodology, W.S. and M.R.; investigation, D.B., J.B., O.K., R.T., W.S., and M.R.; writing – original draft, M.F., W.S., and M.R.; writing – review and editing, W.S. and M.R.; funding acquisition, W.S. and M.R.; resources, K.S., S.S., M.F., and H.H.S.; supervision, W.S. and M.R.

## Declaration of interests

The authors declare no competing interests.

## STAR★Methods

### Key resources table

**Table:** 

REAGENT or RESOURCE	SOURCE	IDENTIFIER
**Antibodies**		

Anti-HA	Sigma-Aldrich	RRID: AB_390918
Anti- myc	Sigma-Aldrich	RRID: AB_568800
Anti-b-tubulin	Cell Signaling Technologies	RRID: AB_2715541
Anti-GAPDH	Abcam	RRID: AB_2687666
Anti-mouse IgG (H + L)-HRP	Bio-Rad	RRID: AB_2617113
Anti-rabbit IgG (H + L)-HRP	Bio-Rad	RRID: AB_11125142
Anti-rat IgG (H + L)-HRP	Thermo Fisher Scientific	RRID: AB_228356

**Bacterial and virus strains**		

DH5α competent E.coli	Thermo Fisher Scientific	Cat # 16512160

**Chemicals, peptides, and recombinant proteins**		

Doxycycline	Cayman Chemical	#CAY14422-1
Bafilomycin A	Aviva System Biology	#OOMC00028-100UG
MG132	Cayman Chemical	#CAY13697-1
Geneticin	Capricorn Scientific	#G418-B
Lipofectamine 3000	Thermo Fisher Scientific	N/A
PNGase F	NEB	#P0704S
EndoH	NEB	#P0702S
CellMask Deep Red Plasma Membrane Stain	Thermo Fisher Scientific	#C10046
Creatine	Sigma-Aldrich	#C3630
[^3^H]creatine	Hartmann Analytic GmbH	#ART1130

**Critical commercial assays**		

Luna Universal One-Step RT-qPCR Kit	NEB	#E3005
RNeasy Mini Kit	Qiagen	#74104
CloneAmp HiFi PCR Premix kit	Takara	#639298
Gateway LR Clonase II Enzyme mix	Invitrogen	#11791020
Gateway BP Clonase II Enzyme mix	Invitrogen	#11789020

**Experimental models: Cell lines**		

Human: HEK293	Prof. Harald SItte (MUW)	N/A
Human: HEK293 Lenti-X	Takara	Cat # 632180

**Oligonucleotides**		

DNA primers for cloning, gRNAs, qPCR primers (see Tables S1–S3)	Integrated DNA Technologies	N/A

**Recombinant DNA**		

pΔ8.9	Addgene	Cat # 187441
pCMV-VSV-G	Addgene	Cat # 8454
pInducer20	Addgene	Cat # 44012
pDonR221	Addgene	Cat # 12536017
peGFP-N1	Clontech	N/A
pDonR221-SLC6A8	Addgene	Cat # 131915
pDonR221-mCherry	Dr. Marko Roblek (MUW)	N/A
pDonR221-SLC16A12	Addgene	Cat # 161124
Px458eCas9WT-2A-dTomate	Feng Zhang lab	N/A

**Software and algorithms**		

FlowJo v10 software	Tree Star Inc.	RRID: SCR_008520
GraphPad Prism 10	GraphPad Software	RRID: SCR_002798
ImageJ	NIH	RRID: SCR_003070
Clampfit software	Molecular Devices	RRID: SCR_011323
Genetic fitting algorithm	Zenodo	https://doi.org/10.5281/zenodo.21626572

### Experimental model and study participant details

#### Cell lines

HEK293, HEK293 eGFP-P2A-K_n_-mCherry, HEK293 mCherry-P2A-K_n_-eGFP, HEK293-SLC6A8^−/−^, HEK293-SLC6A8^−/−^ CRT1^3xHA^-P2A-K_n_-MCT12^2xmyc^, HEK293-SLC6A8^−/−^ CRT1^3xHA^, HEK293-SLC6A8^−/−^ MCT12^2xmyc^, HEK293-SLC6A8^−/−^ CRT1^eGFP^-P2A-K_0_- MCT12^mCherry^, HEK293-SLC6A8^−/−^ MCT12^mCherry^-P2A-K_0_- CRT1^eGFP^, HEK293 Lenti-X (Takara, #632180, Kusatsu, Japan) were cultured in DMEM (Capricorn Scientific, #HXRXA, Ebsdorfergrund, Germany) supplemented with 10% FCS (Capricorn, #FBS-11A), stable glutamine (Capricorn Scientific, #STA-B) and incubated at 37°C with 5% CO2. Expression of recombinant proteins was induced for 24 h prior to experiments using either 100 ng/mL for cellular uptake experiments, or 1 μg/mL for flow cytometry, live-cell imaging, whole-cell patch-clamping and Immunoblot analysis doxycycline (Cayman Chemical, #CAY14422-1, Ann Arbor, USA). Proteolytic degradation was blocked by incubation with 80 nM bafilomycin A (Aviva System Biology, #OOMC00028-100UG, San Diego, USA) and 10 μM MG132 (Cayman Chemical, #CAY13697-1) for 24 h.

The cell lines were not authenticated. All cell lines were routinely tested for mycoplasma infection using Mycoplasma PCR Detection Kit (abm, #G238, Richmond, Canada).

### Method details

#### Lentiviral infection

Lentiviral supernatant was prepared by co-transfection of pΔ8.9 (Addgene, #187441) and pCMV-VSV-G (Addgene, #8454) with the expression plasmid pInducer20^35^ into Lenti-X 293T cells (TaKaRa, #632180). The supernatant was harvested after 48 h and used to infect target HEK293 or HEK293 SLC6A8^−/−^ cells. Cells, which stably integrated the expression construct were selected with 2 mg/mL geneticin (G418, Capricorn Scientific, #G418-B) for one week.

#### Generation of HEK293 SLC6A8^−/−^

HEK293 cells were transfected with two px458eCas9WT-2A-dTomato (Feng Zhang lab) plasmids expressing two SLC6A8 specific gRNAs (Table S1) using Lipofectamine 3000 (ThermoFisher Scientific, #L3000008, Waltham, USA) according to the manufacturer’s instructions. One day after transfection, dTomato^+^ cells were sorted by flow cytometry using a BD ARIAFusion flow cytometer (Becton Dickinson, Franklin Lakes, USA). On the following day, cells were seeded into 96-well plates under limiting dilution conditions. Two weeks later, single cell clones were identified by visual inspection with a 4*x*/0.10 objective using a Nikon ECLIPSE Ts2 microscope (Nikon, Tokyo, Japan) and expanded. The deletion of SLC6A8 was verified by radioligand uptake experiments.

#### qPCR analysis

qPCR was performed with the Luna Universal One-Step RT-qPCR Kit (NEB, #E3005) using primers listed in Table S2 and an input of 0.1 μg–1.0 μg of total isolated RNA (RNeasy Mini Kit, Qiagen, #74104, Hilden, Germany) on a CFX Connect machine (Bio-Rad, Hercules, USA).

#### Construct cloning

PCR reactions were performed with the CloneAmp HiFi PCR Premix kit (Takara, #639298) using primers listed in Table S3, and all constructs were assembled via PCR. PCR amplified constructs were cloned by Gateway cloning (ThermoFisher Scientific) using the donor plasmid pDONR221 (Addgene, #12536017) into the doxycycline-inducible expression plasmid pInducer20.^35^ All constructs were introduced into DH5α *E. coli* by heat-shock transformation. The correct sequence of all constructs was confirmed by whole-plasmid sequencing (Plasmidsaurus, South San Francisco, USA) (Table S4). All primers were from IDT (Integrated DNA Technologies, Coralville, USA).

#### Immunoblotting

Cell lysates were separated on 4%–15% SDS-PAGE gradient gels (Bio-Rad, Hercules, USA) and transferred onto Protran 0.45 nitrocellulose membranes (GE Healthcare, Chicago, USA). The nitrocellulose membrane was blocked with SuperBlock Blocking Buffer (ThermoFisher Scientific, #37535) for 15 min at room temperature and incubated with primary antibodies in 1 *x* Pierce Clear Milk Blocking Buffer (ThermoFisher Scientific, #37587) overnight at 4°C. The incubation with secondary antibodies was done in 1 *x* Pierce Clear Milk Blocking Buffer for 1 h at room temperature. Following washing with PBS, membranes were incubated with SuperSignal West Pico PLUS Chemiluminescent Substrate (ThermoFisher Scientific, #34577), and the chemiluminescent signal was detected with the ChemiDox MP Gel Imaging System (Bio-Rad). Antibodies used are listed in Table S5.

Enzymatic deglycosylation was performed with peptide-N-glycosidase F (PNGase F; NEB, #P0704S, Ipswich, USA) and endoglycosidase H (EndoH; NEB, #P0702S) according to the manufacturer’s instructions.

#### Live-cell imaging

Cells were grown on poly-D-lysine-coated glass-bottom dishes (Cellvis, #D35-20-1.5-N, Mountain View, USA). Expression of SLC6A8^eGFP^-P2A-K_0_-SLC16A12^mCherry^ or SLC16A12^mCherry^-P2A-K_0_-SLC6A8^eGFP^ was induced for 24 h with 1 μg/mL doxycycline. Prior to confocal imaging, cells were rinsed once in Live cell imaging solution (Gibco, #A59688DJ) and the cell membrane was stained with CellMask Deep Red Plasma Membrane stain (Thermo Fisher Scientific, #C10046) at 1:2000 dilution in Live cell imaging solution for 3 min. Cells were rinsed thrice with the imaging solution, and confocal images were captured on a Nikon Eclipse Ti (Nikon) confocal microscope with a Plan-Apochromat VC *x*60/NA 1.4 Oil DIC N2 objective. Images were processed by ImageJ.^36^ The plasma membrane staining served as a region of interest for detection of membrane-bound SLC6A8^eGFP^ and SLC16A12^mCherry^. The relative membrane localization of the transporters was determined by calculating the ratio of the fluorescence in the membrane over the total cellular fluorescence.

#### Whole-cell patch-clamping

HEK293 cells were grown on poly-D-lysine-coated cell culture dishes. Protein expression was induced by incubation of cells for 24 h in the presence of 1 μg/mL doxycycline prior to recordings. During experiments, HEK293 cells were maintained in a bath solution consisting of 150 mM NaCl, 3 mM KCl, 2.5 mM CaCl_2_, 2 mM MgCl_2_, 20 mM glucose, and 10 mM HEPES, with the pH adjusted to pH 7.4 using NaOH.

The patch pipette solution contained 150 mM potassium methanesulfonate, 1 mM CaCl_2_, 0.7 mM MgCl_2_, 10 mM HEPES, and 10 mM EGTA. The pH was adjusted to pH 7.2 with methanesulfonic acid.

All buffers were prepared freshly before each experiment. All chemicals were of analytical grade and were used without further purification.

Creatine (Sigma-Aldrich, #C3630, St. Louis, USA) was applied via a perfusion system (Octaflow II, ALA Scientific Instruments Inc., Farmingdale, USA), which allowed for rapid and complete solution exchange around the cells within 20 ms.

Experiments were conducted with precise temperature control provided by a temperature control unit (cell microcontrol inline preheater, Green Leaf Scientific, Dublin, Ireland), which was linked to a temperature control system (PTC-20, npi electronic GmbH, Tamm, Germany). Both, the patch-clamp pipette, and the perfusion system were manipulated using the PatchStar micromanipulator (Scientifica Ltd., Uckfield, East Sussex, UK).

Patch clamp recordings were done in the whole-cell configuration using an Axon 200B amplifier equipped with an Axon 1550 digitizer (both from Molecular Devices, LLC, San Jose, USA). Recordings were sampled at 10 kHz. The current amplitudes were quantified using Clampfit software (Molecular Devices, LLC). Passive holding currents were subtracted, and the traces were digitally filtered using an 80 Hz digital 8-pole Bessel low-pass filter.

#### Cellular uptake of [^3^H]creatine

HEK293 cells (10000) were seeded on poly-D-lysine-coated 96-well plates (Sarstedt, #83.3924, Nümbrecht, Germany) 24 h prior to uptake experiments.

Expression of varying ratios of CRT1 to MCT12 (K_0_–K_6_) was induced with 100 ng/mL doxycycline for 24 h and cells were then exposed to 10 nM [^3^H]creatine (specific activity of 75 Ci/mmol, Hartmann Analytic GmbH, Braunschweig, Germany) at 37°C in DMEM medium devoid of fetal calf serum. The uptake reaction was terminated after 15, 30, 60, 175, and 375 min by aspiration of the radioactive solution followed by two rapid washes with ice-cold Krebs-HEPES buffer (10 mM HEPES pH 7.4 (adjusted with NaOH), 4.7 mM KCl, 2.2 mM CaCl_2_, 1.2 mM MgSO_4_, 10 mM glucose, 120 mM NaCl). Subsequently, cells were lysed in scintillation cocktail (ROTISZINT®Routine, Carl Roth, Karlsruhe, Germany), and intracellular radioligand was quantified using a liquid scintillation counter (1450 MicroBeta, PerkinElmer Life Sciences, Wallac Oy, Turku, Finland).

#### Flow cytometry

The expression of ectopic proteins was induced with 1 μg/mL doxycycline 24 h prior to analysis. Cells were detached with trypsin (Sigma-Aldrich, #T3924) and resuspended in cold FACS buffer (1% BSA, 10 mM EDTA in PBS). Heterologous expression of fluorescent proteins was analyzed on a Beckman Coulter CytoFLEX S (Beckman Coulter, Brea, USA) flow cytometer. Data were processed by FlowJo software (Tree Star Inc., Ashland, USA).

#### Genetic curve fitting algorithm

This algorithm generates a user-defined number (e.g., 20) of initial sets of rate constants (i.e., entities) where the rate constants are randomly drawn between adjustable upper and lower bounds.^37^ For the set of parameters of each entity, the system of differential equations, which defines the kinetic model, is then solved using the SUNDIALS-CVODE solver.^38^^,^^39^ The “fitness” parameter for an entity is calculated by subtracting the simulated data from the measured data. The resulting difference is then squared and summed to generate the sum of squares for the dataset (SSQ). The parameter set is optimized by a user-defined number of iterations (e.g., 1000) of the following algorithm: (i) Define a number of parent entities (e.g., 10). (ii) Randomly select a user-defined number of entities to compete in a tournament (e.g., 10). The entity with the lowest SSQ in a tournament is selected as a parent. (iii) Repeat step (ii) until the desired number of parents is reached. (iv) Randomly pair two parents and produce an offspring, receiving parameters from both parent entities through random crossover. (v) A user-defined mutation rate determines which rate constants undergo random changes around their original values. (vi) Replace a subset of entities, selected via tournament selection for the highest SSQ with the new offspring and proceed with step (i).

### Quantification and statistical analysis

Experimental errors are stated as standard error of the mean (SEM) or standard deviation (SD), as indicated in the figure legends.

## Supplemental information

Document S1. Figures S1–S3 and Tables S1, S2, and S5

Table S3. Plasmids used in this study

Table S4. Construct sequences used in this study confirmed by whole plasmid sequencing (A) Nucleotide sequence of eGFP-P2A-K_n_-mCherry. (B) Nucleotide sequence of mCherry-P2A-K_n_-eGFP. (C) Nucleotide sequence of SLC6A8^3xHA^-P2A-K_n_-SLC16A12^2xmyc^. (D) Nucleotide sequence of SLC6A8^eGFP^-P2A-K_0_-SLC16A12^mCherry^. (E) Nucleotide sequence of SLC16A12^mCherry^-P2A-K_0_-SLC6A8^eGFP^. (F) Nucleotide sequence of SLC6A8^3xHA^. (G) Nucleotide sequence of SLC16A12^2xmyc^.

## References

[1] Wigler M., Sweet R., Sim G.K., Wold B., Pellicer A., Lacy E., Maniatis T., Silverstein S., Axel R. (1979). Transformation of mammalian cells with genes from procaryotes and eucaryotes. Cell.

[2] Smith E.M., Mueller J.D. (2012). The statistics of protein expression ratios for cellular fluorescence studies. Eur. Biophys. J..

[3] Pilbrough W., Munro T.P., Gray P. (2009). Intraclonal protein expression heterogeneity in recombinant CHO cells. PLoS One.

[4] Baliga U.K., Gurunian A., Nogales A., Martinez-Sobrido L., Dean D.A. (2025). Electroporation- and Liposome-Mediated Co-Transfection of Single and Multiple Plasmids. Pharmaceutics.

[5] Di Blasi R., Marbiah M.M., Siciliano V., Polizzi K., Ceroni F. (2021). A call for caution in analysing mammalian co-transfection experiments and implications of resource competition in data misinterpretation. Nat. Commun..

[6] Liu Z., Chen O., Wall J.B.J., Zheng M., Zhou Y., Wang L., Vaseghi H.R., Qian L., Liu J. (2017). Systematic comparison of 2A peptides for cloning multi-genes in a polycistronic vector. Sci. Rep..

[7] Dai W., Vinnakota S., Qian X., Kunze D.L., Sarkar H.K. (1999). Molecular characterization of the human CRT-1 creatine transporter expressed in Xenopus oocytes. Arch. Biochem. Biophys..

[8] Abplanalp J., Laczko E., Philp N.J., Neidhardt J., Zuercher J., Braun P., Schorderet D.F., Munier F.L., Verrey F., Berger W. (2013). The cataract and glucosuria associated monocarboxylate transporter MCT12 is a new creatine transporter. Hum. Mol. Genet..

[9] Farr C.V., El-Kasaby A., Erdem F.A., Sucic S., Freissmuth M., Sandtner W. (2022). Cooperative Binding of Substrate and Ions Drives Forward Cycling of the Human Creatine Transporter-1. Front. Physiol..

[10] Koutmou K.S., Schuller A.P., Brunelle J.L., Radhakrishnan A., Djuranovic S., Green R. (2015). Ribosomes slide on lysine-encoding homopolymeric A stretches. eLife.

[11] Arthur L., Pavlovic-Djuranovic S., Smith-Koutmou K., Green R., Szczesny P., Djuranovic S. (2015). Translational control by lysine-encoding A-rich sequences. Sci. Adv..

[12] Charneski C.A., Hurst L.D. (2013). Positively charged residues are the major determinants of ribosomal velocity. PLoS Biol..

[13] Futagi Y., Narumi K., Furugen A., Kobayashi M., Iseki K. (2020). Molecular characterization of the orphan transporter SLC16A9, an extracellular pH- and Na(+)-sensitive creatine transporter. Biochem. Biophys. Res. Commun..

[14] Erdem F.A., Ilic M., Koppensteiner P., Gołacki J., Lubec G., Freissmuth M., Sandtner W. (2019). A comparison of the transport kinetics of glycine transporter 1 and glycine transporter 2. J. Gen. Physiol..

[15] King E.L., Altman C. (1956). A Schematic Method of Deriving the Rate Laws for Enzyme-Catalyzed Reactions. J. Phys. Chem..

[16] Schicker K., Bhat S., Farr C., Burtscher V., Horner A., Freissmuth M., Sandtner W. (2021). Descriptors of Secondary Active Transporter Function and How They Relate to Partial Reactions in the Transport Cycle. Membranes.

[17] Juszkiewicz S., Hegde R.S. (2017). Initiation of Quality Control during Poly(A) Translation Requires Site-Specific Ribosome Ubiquitination. Mol. Cell.

[18] Garshott D.M., An H., Sundaramoorthy E., Leonard M., Vicary A., Harper J.W., Bennett E.J. (2021). iRQC, a surveillance pathway for 40S ribosomal quality control during mRNA translation initiation. Cell Rep..

[19] Mattiroli F., Sixma T.K. (2014). Lysine-targeting specificity in ubiquitin and ubiquitin-like modification pathways. Nat. Struct. Mol. Biol..

[20] Guharoy M., Bhowmick P., Sallam M., Tompa P. (2016). Tripartite degrons confer diversity and specificity on regulated protein degradation in the ubiquitin-proteasome system. Nat. Commun..

[21] Chandrasekaran V., Juszkiewicz S., Choi J., Puglisi J.D., Brown A., Shao S., Ramakrishnan V., Hegde R.S. (2019). Mechanism of ribosome stalling during translation of a poly(A) tail. Nat. Struct. Mol. Biol..

[22] Tesina P., Lessen L.N., Buschauer R., Cheng J., Wu C.C.C., Berninghausen O., Buskirk A.R., Becker T., Beckmann R., Green R. (2020). Molecular mechanism of translational stalling by inhibitory codon combinations and poly(A) tracts. EMBO J..

[23] Lu J., Deutsch C. (2008). Electrostatics in the ribosomal tunnel modulate chain elongation rates. J. Mol. Biol..

[24] Farr C.V., El-Kasaby A., Freissmuth M., Sucic S. (2020). The Creatine Transporter Unfolded: A Knotty Premise in the Cerebral Creatine Deficiency Syndrome. Front. Synaptic Neurosci..

[25] Wells C., Sorgenfrei J., Johnson S.L., Albertson D., Rutter J., Baker S.A. (2025). Gene delivery of AGAT and GAMT boosts creatine levels in creatine transporter deficiency patient fibroblasts. PLoS One.

[26] Stimpson S.A., Turner S.M., Clifton L.G., Poole J.C., Mohammed H.A., Shearer T.W., Waitt G.M., Hagerty L.L., Remlinger K.S., Hellerstein M.K., Evans W.J. (2012). Total-body creatine pool size and skeletal muscle mass determination by creatine-(methyl-D3) dilution in rats. J. Appl. Physiol..

[27] Takahashi M., Kishimoto H., Shirasaka Y., Inoue K. (2020). Functional characterization of monocarboxylate transporter 12 (SLC16A12/MCT12) as a facilitative creatine transporter. Drug Metab. Pharmacokinet..

[28] Braissant O., Henry H. (2008). AGAT, GAMT and SLC6A8 distribution in the central nervous system, in relation to creatine deficiency syndromes: a review. J. Inherit. Metab. Dis..

[29] Kushmerick M.J., Moerland T.S., Wiseman R.W. (1992). Mammalian skeletal muscle fibers distinguished by contents of phosphocreatine, ATP, and Pi. Proc. Natl. Acad. Sci. USA.

[30] Rackayova V., Cudalbu C., Pouwels P.J.W., Braissant O. (2017). Creatine in the central nervous system: From magnetic resonance spectroscopy to creatine deficiencies. Anal. Biochem..

[31] Wyss M., Kaddurah-Daouk R. (2000). Creatine and creatinine metabolism. Physiol. Rev..

[32] Wallis J., Lygate C.A., Fischer A., ten Hove M., Schneider J.E., Sebag-Montefiore L., Dawson D., Hulbert K., Zhang W., Zhang M.H. (2005). Supranormal myocardial creatine and phosphocreatine concentrations lead to cardiac hypertrophy and heart failure: insights from creatine transporter-overexpressing transgenic mice. Circulation.

[33] Uhlén M., Fagerberg L., Hallström B.M., Lindskog C., Oksvold P., Mardinoglu A., Sivertsson Å., Kampf C., Sjöstedt E., Asplund A. (2015). Proteomics. Tissue-based map of the human proteome. Science.

[34] Flögel S., Strater M., Fischer D., Gründemann D. (2025). A creatine efflux transporter in oligodendrocytes. FEBS J..

[35] Meerbrey K.L., Hu G., Kessler J.D., Roarty K., Li M.Z., Fang J.E., Herschkowitz J.I., Burrows A.E., Ciccia A., Sun T. (2011). The pINDUCER lentiviral toolkit for inducible RNA interference in vitro and in vivo. Proc. Natl. Acad. Sci. USA.

[36] Schneider C.A., Rasband W.S., Eliceiri K.W. (2012). NIH Image to ImageJ: 25 years of image analysis. Nat. Methods.

[37] Petrowski A., Ben-Hamida S. (2017). Evolutionary Algorithms.

[38] Hindmarsh A.C., Brown P.N., Grant K.E., Lee S.L., Serban R., Shumaker D.E., Woodward C.S. (2005). SUNDIALS: Suite of nonlinear and differential/algebraic equation solvers. ACM Trans. Math. Softw..

[39] Gardner D.J., Reynolds D.R., Woodward C.S., Balos C.J. (2022). Enabling New Flexibility in the SUNDIALS Suite of Nonlinear and Differential/Algebraic Equation Solvers. ACM Trans. Math. Softw..

