# Supplementary material for: A method for expressing two proteins at a tunable ratio allows for studying the kinetics of creatine transport by MCT12
Source: iScience. 2026 Sep 30;29(10):117719. doi: 10.1016/j.isci.2026.117719 (PMC13657109; doi:10.1016/j.isci.2026.117719)

iScience, Volume *29*

Supplemental information

A method for expressing two proteins at a tunable ratio allows for studying the kinetics of creatine transport by MCT12

Danila Boytsov, Julia Bicher, Oliver Kudlacek, Klaus Schicker, Rodion Tsurikov, Sonja Sucic, Michael Freissmuth, Harald H. Sitte, Walter Sandtner, and Marko Roblek

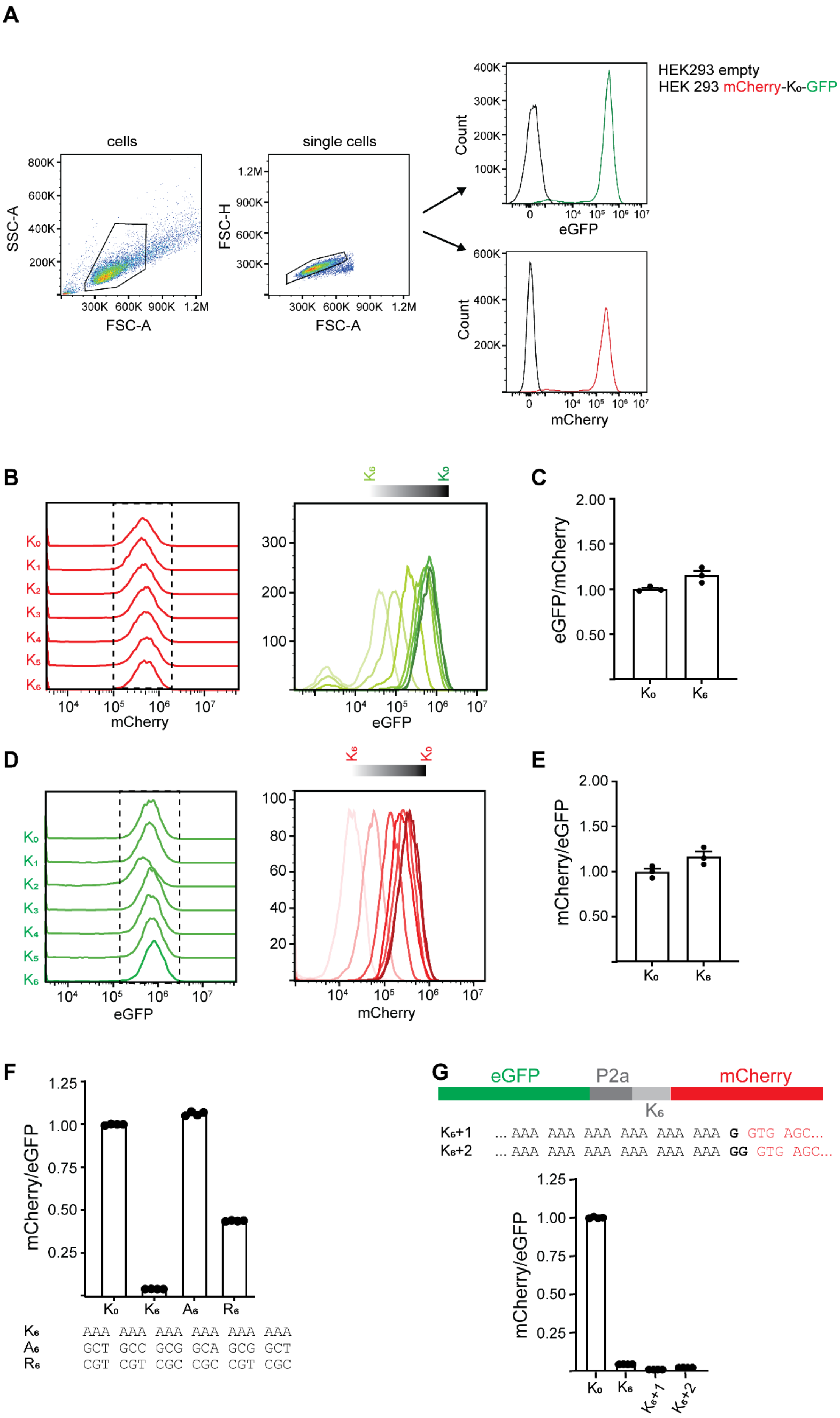
A
cells
SSC-A
FSC-A
single cells
FSC-H
FSC-A
Count
eGFP
Count
mCherry
HEK293 empty
HEK 293 mCherry-K0-GFP
B
K0
K1
K2
K3
K4
K5
K6
mCherry
K6
K0
eGFP
C
eGFP/mCherry
K0
K6
D
K0
K1
K2
K3
K4
K5
K6
eGFP
K6
K0
mCherry
E
mCherry/eGFP
K0
K6
F
mCherry/eGFP
K0
K6
A6
R6
K6 AAA AAA AAA AAA AAA AAA
A6 GCT GCC GCG GCA GCG GCT
R6 CGT CGT CGC CGC CGT CGC
G
eGFP
P2a
mCherry
K6
K6+1 ... AAA AAA AAA AAA AAA AAA G GTG AGC...
K6+2 ... AAA AAA AAA AAA AAA AAA GG GTG AGC...
mCherry/eGFP
K0
K6
K6+1
K6+2

Figure S1. Adaptable expression ratios of two proteins from one open reading frame, related to Figure 1.

A) Flow cytometry gating strategy of HEK293 eGFP-P2A-K_0-mCherry cells is shown. Cells were first gated according to FSC-A and SSC-A. The indicated population of cells was gated for single cells according to FSC-A and FSC-H. The expression level of eGFP and mCherry is shown for HEK293 eGFP-P2A-K_0-mCherry and control HEK293 single cells.

B) Histogram of mCherry and eGFP expression in HEK293 cells harboring the indicated mCherry-P2A-K_n-eGFP constructs was determined by flow cytometry.

C) qPCR analysis of mRNA encoding eGFP and mCherry in HEK293 cells harboring mCherry-P2A-K_0-eGFP and mCherry-P2A-K_6-eGFP. The ratio of mRNAs encoding eGFP and mCherry is shown (n=3).

D) Histogram of eGFP and mCherry expression in HEK293 cells harboring the indicated eGFP-P2A-K_n-mCherry constructs was determined by flow cytometry.

E) qPCR analysis of mRNA encoding eGFP and mCherry in HEK293 cells harboring eGFP-P2A-K_0-mCherry and eGFP-P2A-K_6-mCherry. The ratio of mRNAs encoding mCherry and eGFP expression is shown (n=3).

F) Flow cytometry analysis of HEK293 cells expressing eGFP-P2A-K_0-mCherry, eGFP-P2A-K_6-mCherry, eGFP-P2A-A_6-mCherry, and eGFP-P2A-R_6-mCherry. The ratio of mCherry/eGFP expression is shown (n=4).

G) Scheme of the eGFP-P2A-K_6-eGFP +1 and eGFP-P2A-K_6-eGFP +2 construct, with the nucleotide sequence shown. The ratio of mCherry and eGFP expression was determined by flow cytometry and is displayed in the bottom graph (n=4).

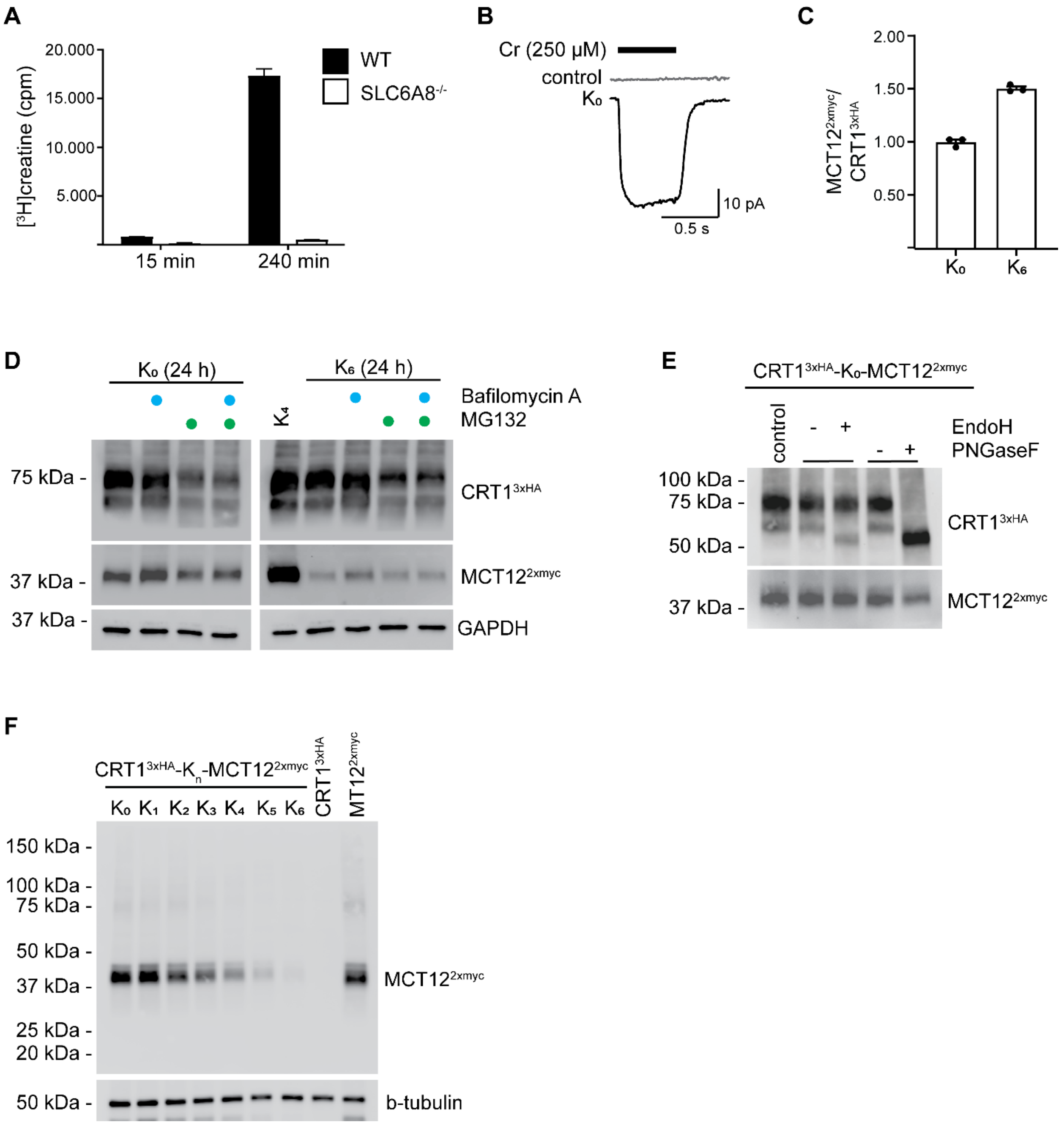

A
[3H]creatine (cpm)
20.000
15.000
10.000
5.000
15 min
240 min
WT
SLC6A8-/-
B
Cr (250 µM)
control
K0
10 pA
0.5 s
C
MCT12 2xmyc/ CRT1 3xHA
2.00
1.50
1.00
0.50
K0
K6
D
K0 (24 h)
K6 (24 h)
Bafilomycin A
MG132
K4
75 kDa -
37 kDa -
37 kDa -
CRT1 3xHA
MCT12 2xmyc
GAPDH
E
CRT1 3xHA-K0-MCT12 2xmyc
control
EndoH
PNGaseF
100 kDa -
75 kDa -
50 kDa -
37 kDa -
CRT1 3xHA
MCT12 2xmyc
F
CRT1 3xHA-Kn-MCT12 2xmyc
K0 K1 K2 K3 K4 K5 K6
CRT1 3xHA
MT12 2xmyc
150 kDa -
100 kDa -
75 kDa -
50 kDa -
37 kDa -
25 kDa -
20 kDa -
MCT12 2xmyc
50 kDa -
b-tubulin

Figure S2. Characterization of HEK293 SLC6A8$^{-/-}$ cells, related to Figure 2.

A) [^{3}H]creatine uptake in HEK293 wild-type and SLC6A8$^{-/-}$ cells (cpm = counts per minute).

B) Representative patch clamp recordings obtained from a HEK293 wild-type and a HEK293 CRT1^{3xHA}-P2A-K_0-MCT12^{2xmyc} cell, respectively. 250 µM creatine failed to elicit a current in HEK293 wild-type despite the presence of endogenous CRT1. Based on these data, we conclude that the endogenous expression level of CRT1 is at least by a factor of 100 lower than that reached by overexpression.

C) qPCR analysis of CRT1 and MCT12 mRNA in HEK293 SLC6A8$^{-/-}$ cells harboring CRT1^{3xHA}-P2A-K_0-MCT12^{2xmyc} and CRT1^{3xHA}-P2A-K_6-MCT12^{2xmyc}. The ratio of mRNAs encoding MCT12 and CRT1 is shown. n=3.

D) Immunoblot of lysates prepared from HEK293 SLC6A8$^{-/-}$ cells harboring CRT1^{3xHA}-P2A-K_6-MCT12^{2xmyc}, which had been treated for 24 h with bafilomycin A (80 nM), MG132 (10 µM), or a combination of both.

E) Deglycosylation of CRT1 and MCT12. Lysates were prepared from HEK293 SLC6A8$^{-/-}$ cells harboring CRT1^{3xHA}-P2A-K_0-MCT12^{2xmyc} and treated with EndoH and PNGaseF. The level of deglycosylation was analyzed by immunoblotting.

F) Immunoblot of lysates prepared from HEK293 SLC6A8$^{-/-}$ cells harbouring CRT1^{3xHA}-P2A-K_6-MCT12^{2xmyc}, CRT1^{3xHA}, and MCT12^{2xmyc} is shown. The blot shows that the anti-myc 4A6 antibody, detecting MCT12^{2xmyc}, detects a clear band just above 37 kDa. However, there is no indication for a presence of a CRT1^{3xHA}-P2A-K_6-MCT12^{2xmyc} fusion protein, which would be expected to run at approximately 115 kDa. This indicates a very efficient peptide bond skipping by the used P2A site.

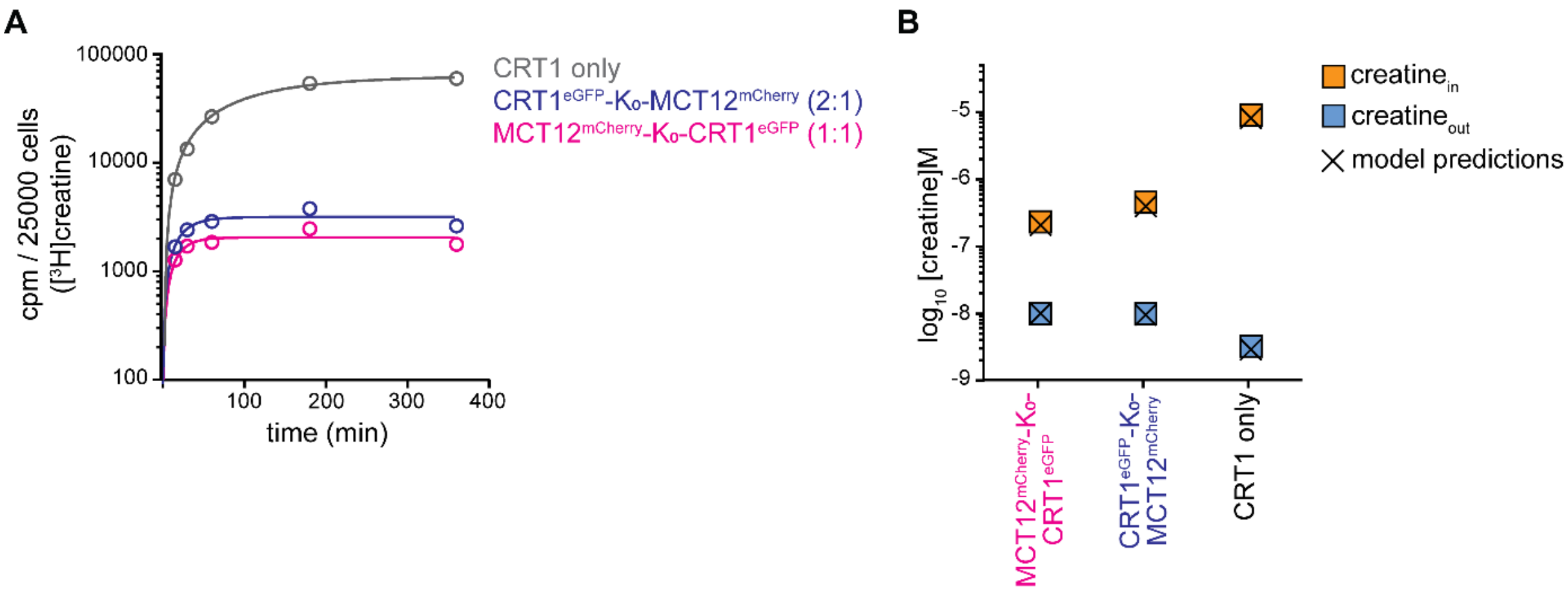

Figure S3. Creatine uptake by CRT1^{eGFP}-P2A-K$_0$-MCT12^{mCherry} and MCT12^{mCherry}-P2A-K$_0$-CRT1^{eGFP}, respectively, related to Figure 4. A) Time-dependent uptake of [3H]creatine (10 nM) into cells expressing the indicated variants and cells expressing only CRT1. **B)** Calculated extra and intracellular creatine concentrations at steady state of the tested constructs together with the corresponding model predictions.

Table S1. gRNA sequences used to generate the HEK293 SLC6A8$^{-/-}$ cell line.

name	sequence
SLC6A8_AD_fw	caccgAATGGGGATTCCTCCAACCA
SLC6A8_AD_rev	aaacTGGTTGGAGGAATCCCCATTc
SLC6A8_1031_fw	caccgCTACAAGGACGCCATCATCC
SLC6A8_1031_rev	aaacGGATGATGGCGTCCTTGTAGc

Table S2. qPCR primer sequences used in this study.

name	sequence
eGFP_fw	AAGGACGACGGCAACTACAA
eGFP_rev	CGATGTTGTGGCGGATCTTG
mCherry_fw	AAGGGCGAGATCAAGCAGAG
mCherry_rev	GCGTTCGTACTGTTCCACGA
GAPDH_fw	CAAGACCTTGGGCTGGGAC
GAPDH_rev	CTGTCGAACAGGAGGAGCAG
SLC6A8_CO_fw	CGTGTATCCTTGGTGGGGAG
SLC6A8_CO_rev	CCACCTTGCTAGACTCGCTC
SLC16A12_CO_fw	CCAAGGTGAACAGGGCAAGA
SLC16A12_CO_rev	TCAGGTGATTGGACACCACG

Table S5. Antibodies used in this study.

Antibody	Clone	Species	Source	Product number
anti-HA	3F10	rat	Sigma-Aldrich	11867432001
anti-myc	4A6	mouse	Sigma-Aldrich	05-724
anti-β-tubulin	D3U1W	mouse	Cell Signaling Technologies	86298T
anti-GAPDH	EPR16884	rabbit	Abcam	ab181603
[redacted]				
anti-mouse IgG (H+L)-HRP		goat	Bio-Rad	1721011
anti-rabbit IgG (H+L)-HRP		goat	Bio-Rad	1706515
anti-rat IgG (H+L)-HRP		goat	Thermo Fisher Scientific	31470

Data S1. Original Western blots corresponding to data presented in Figure 2B-C (n=5).

biological replicate no. 1 and 2

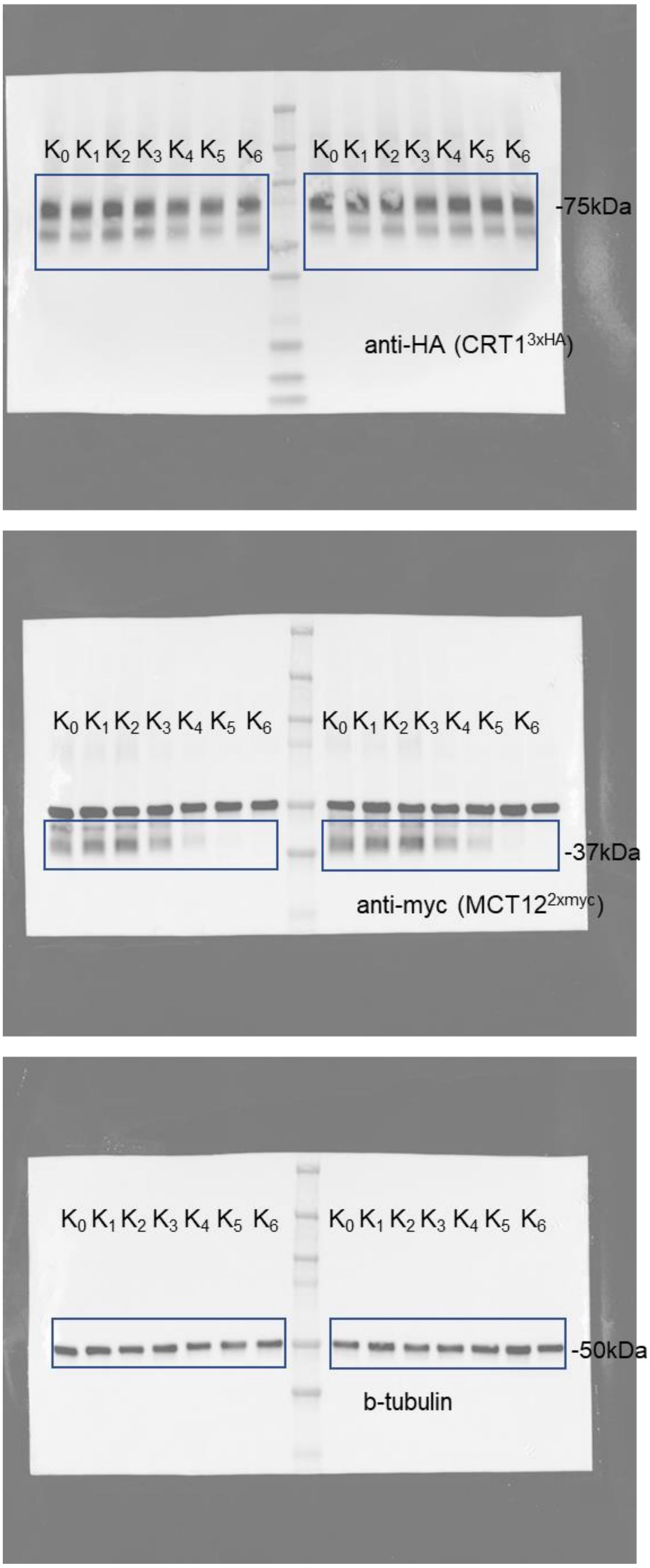

biological replicate no. 3
(used in Figure 3B)

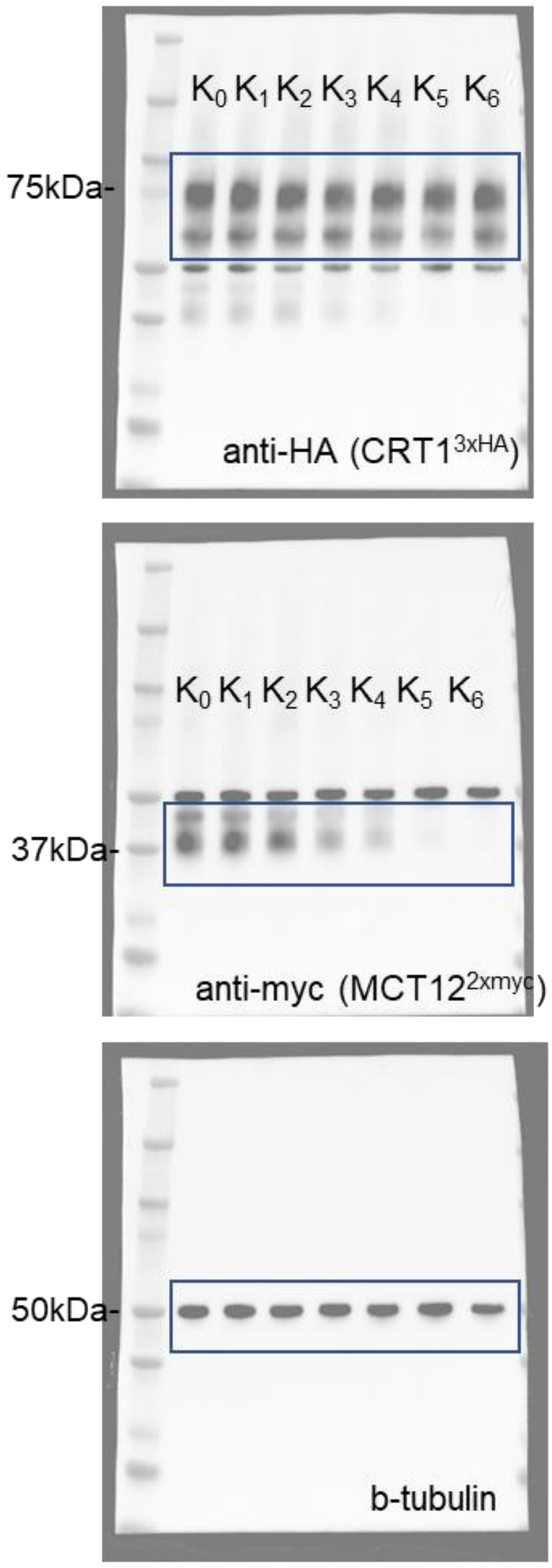

biological replicate no. 4

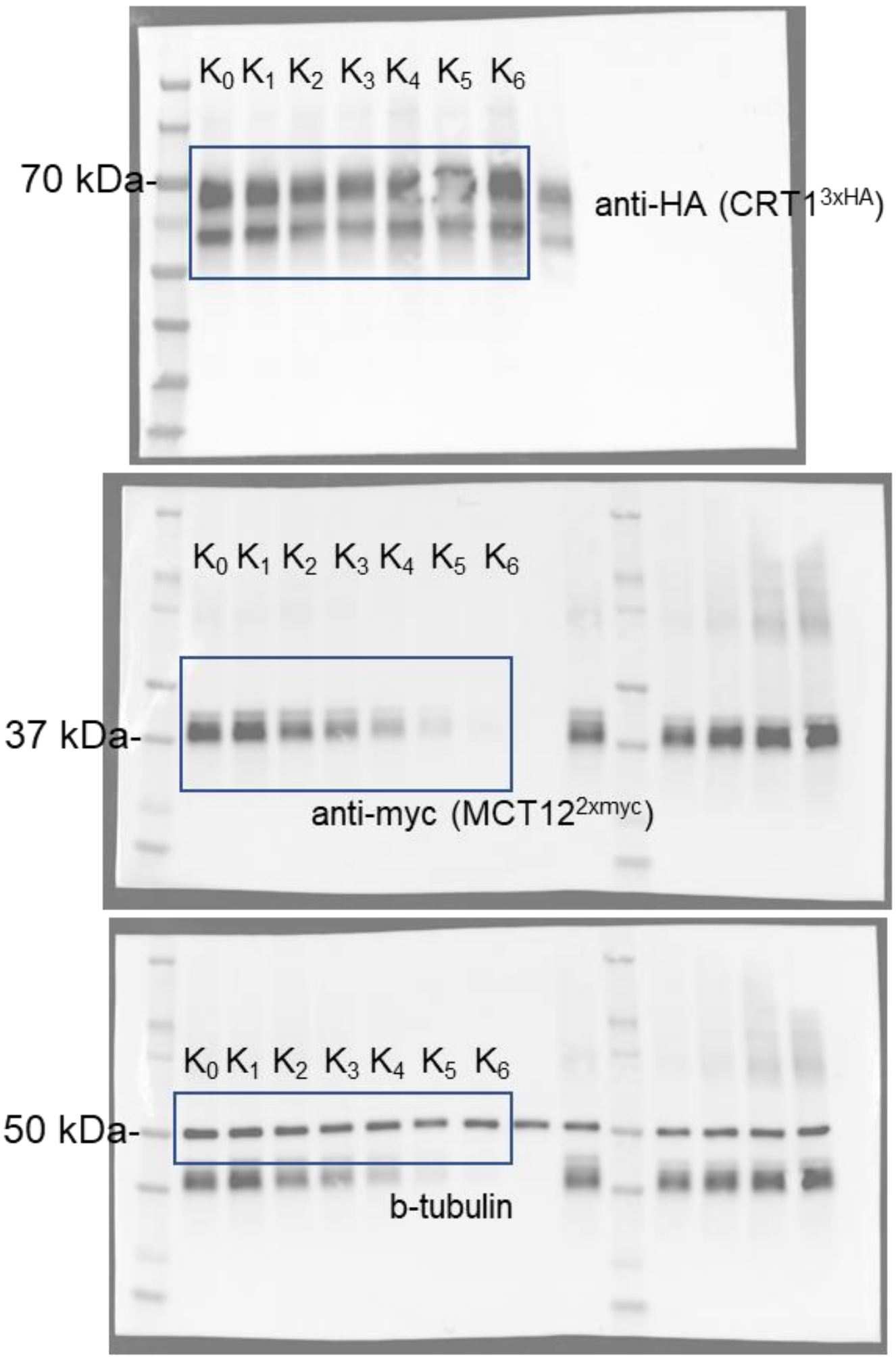

biological replicate no. 5

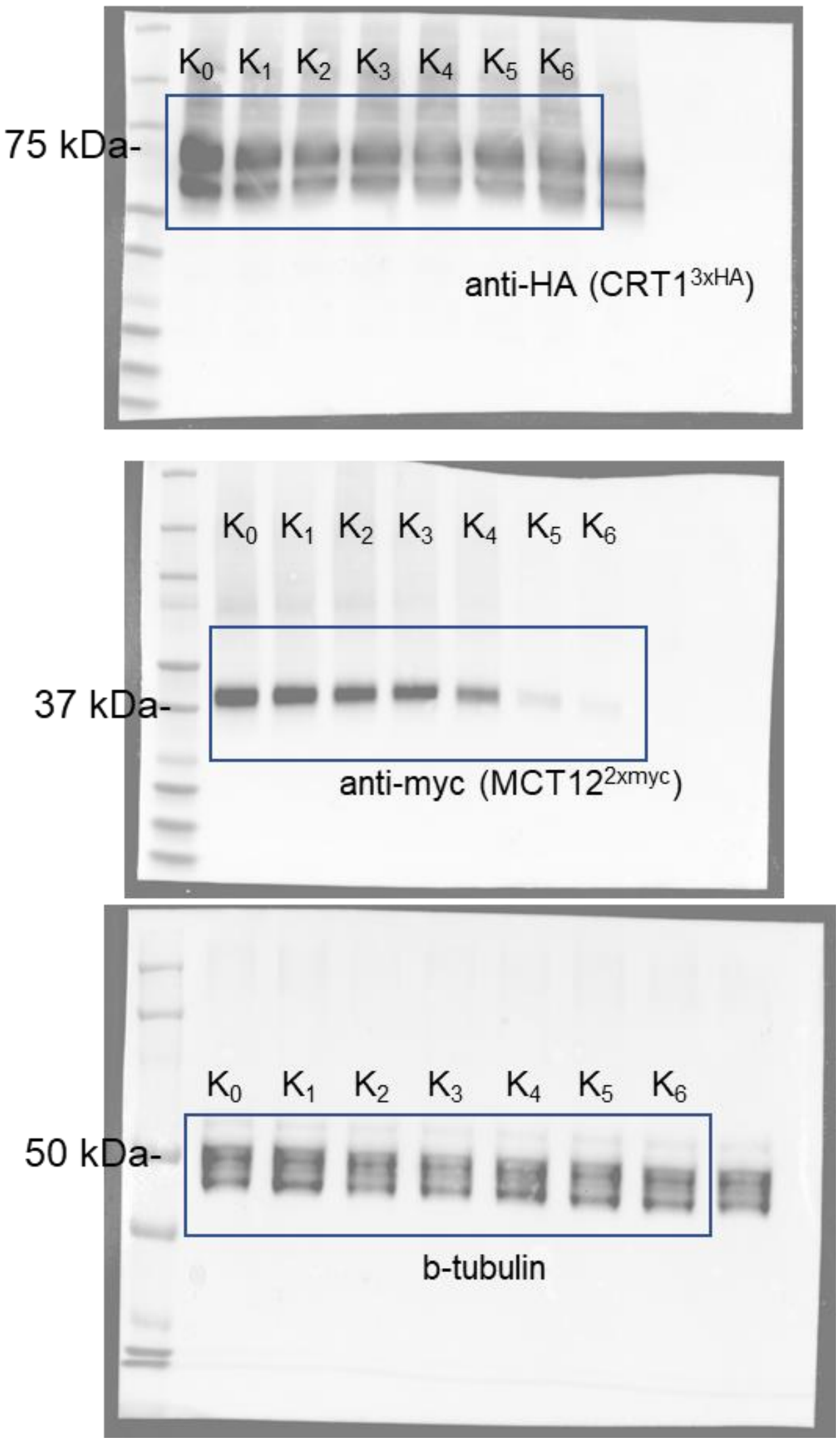

Data S2. Original Western blots corresponding to data presented in Figure 2D-E (n=4).

biological replicate no. 1 and 2

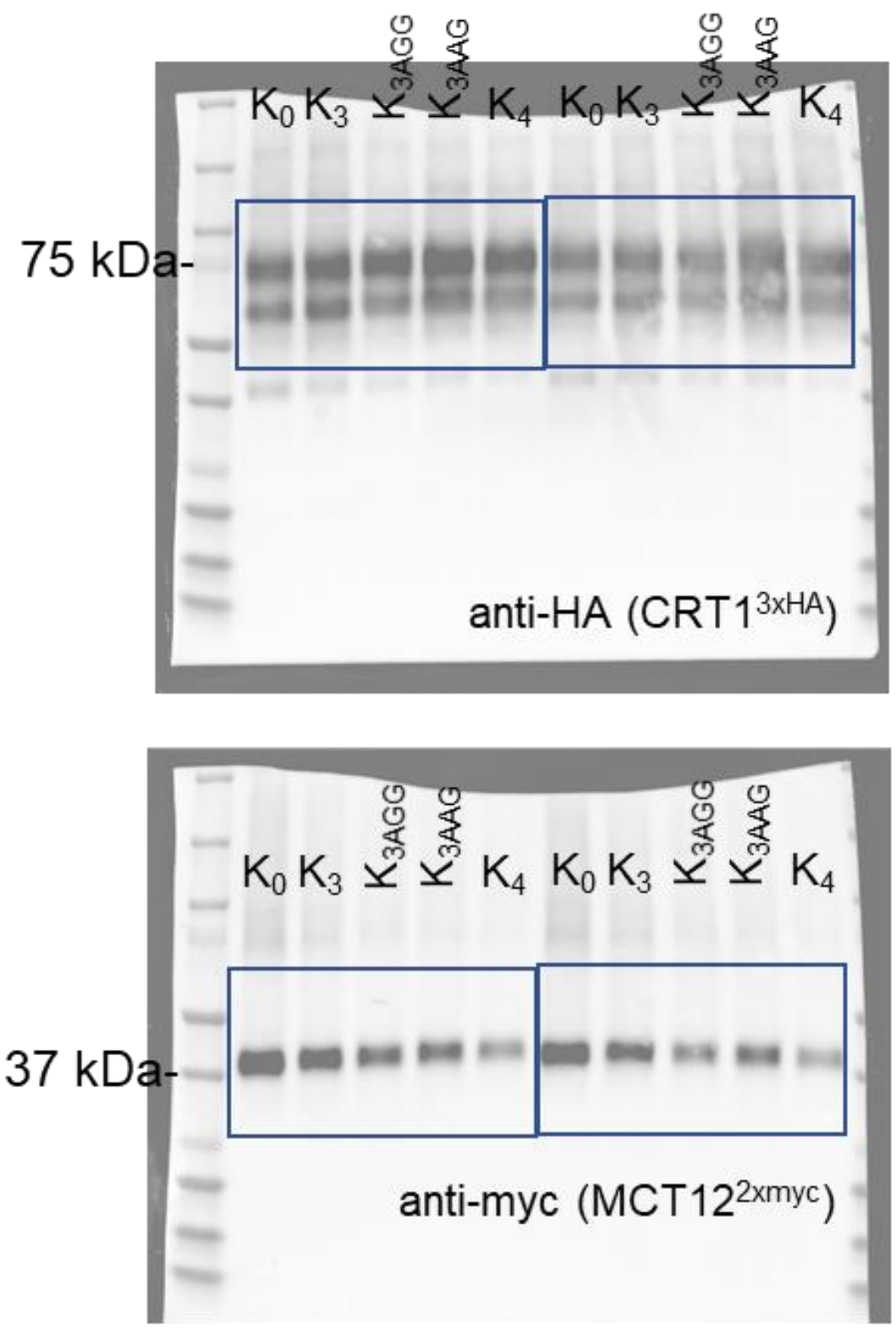

biological replicate no. 3
(used for Figure 3D)

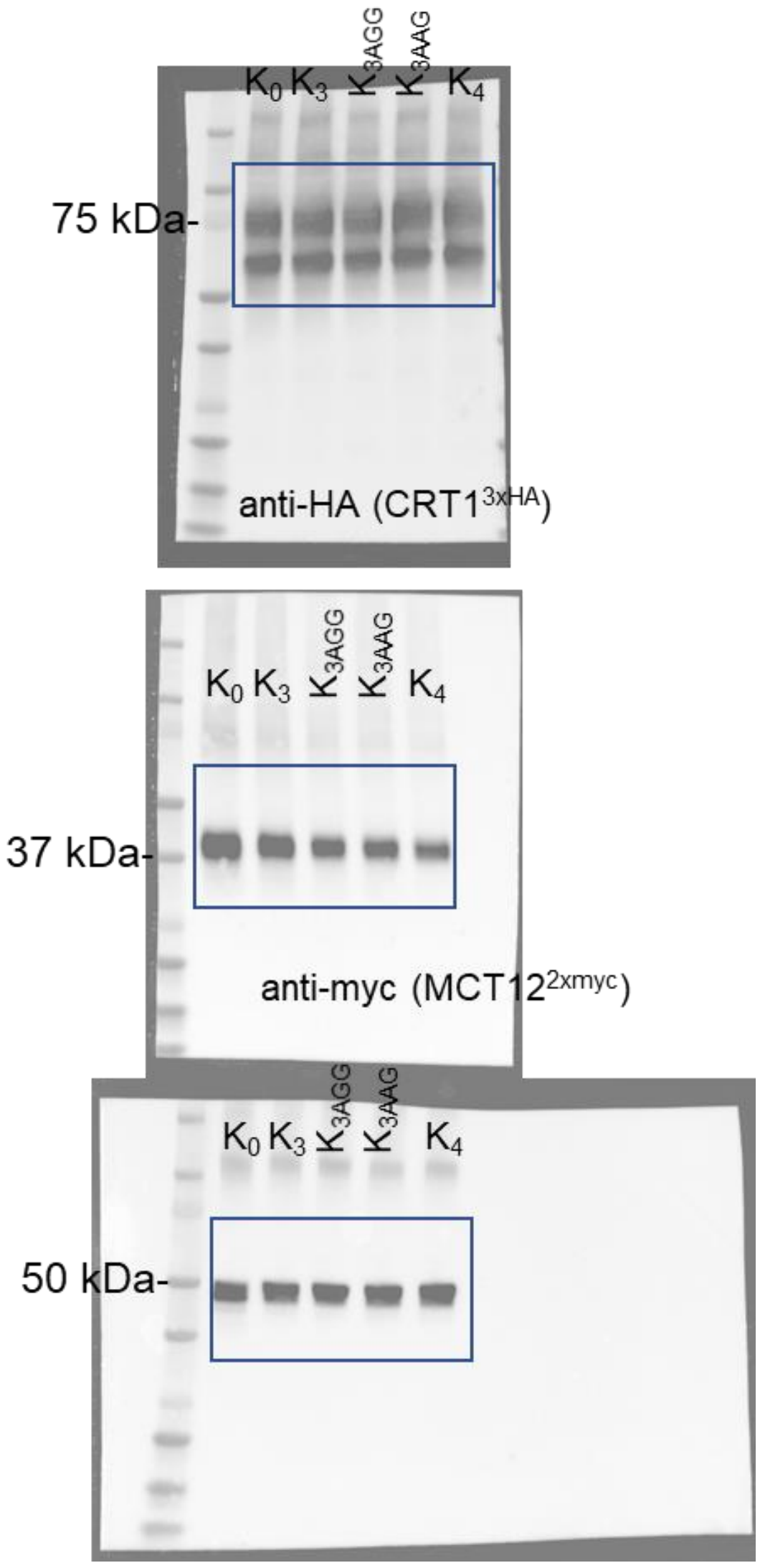

biological replicate no. 4

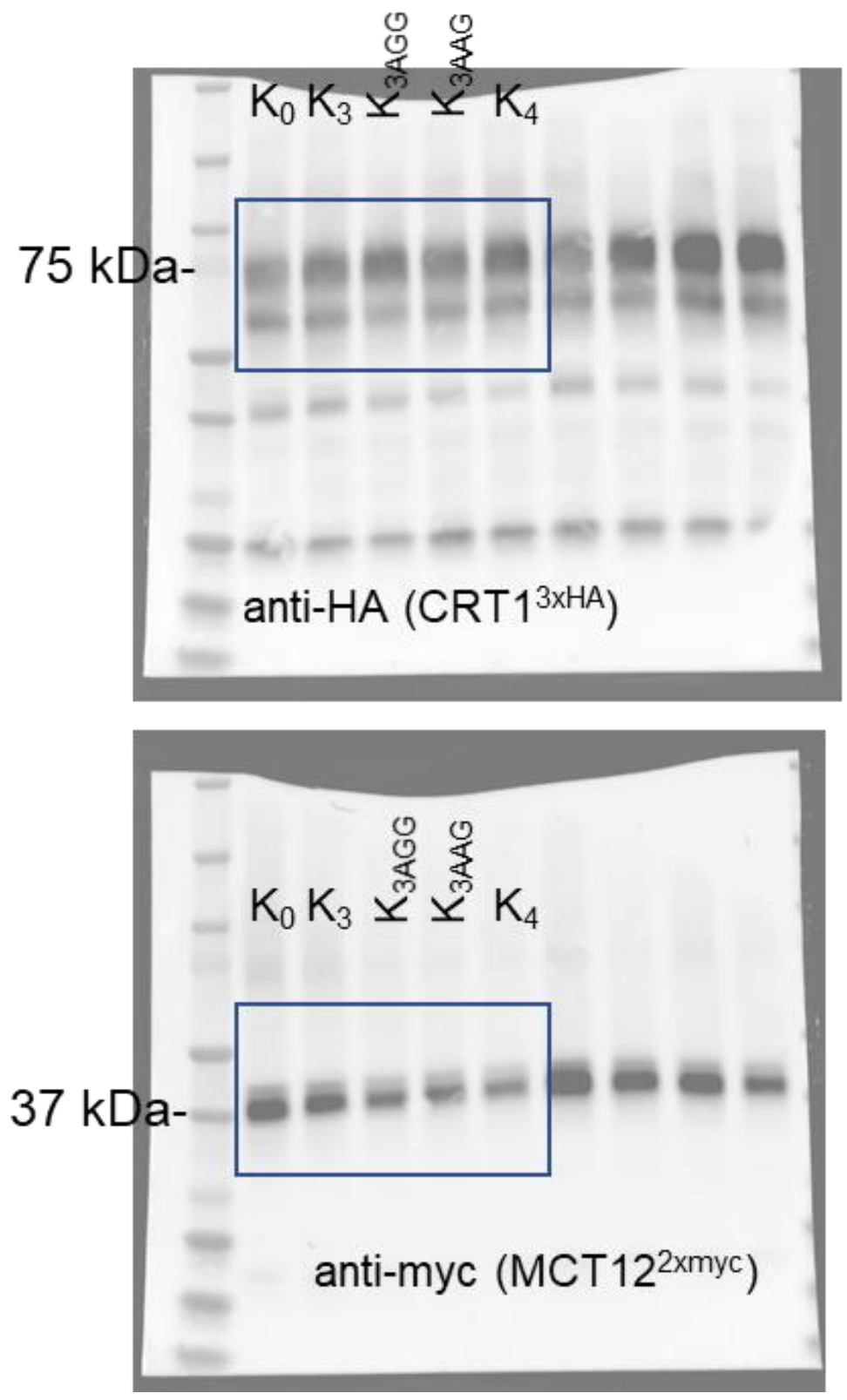

Supplement: Supplementary file 1 [file mmc1.pdf]
